# Barriers and Facilitators to Providing Women Postpartum Care by Health Care Providers in the First 12 Months After Childbirth: A Systematic Qualitative Meta‐Aggregation Review

**DOI:** 10.1111/jmwh.70142

**Published:** 2026-06-16

**Authors:** Maryam Rouhi, Silvana Bettiol, Sarah Prior, Narges Rouhi, Theodora Dedo Azu, Christine Stirling, Lynne Staff, Linda Jones, Antigoni Leftherioti, Patricia Nicholson

**Affiliations:** ^1^ School of Nursing College of Health and Medicine University of Tasmania Hobart Tasmania Australia; ^2^ School of Medicine College of Health and Medicine University of Tasmania Hobart Tasmania Australia; ^3^ School of Medicine Rural Clinical School College of Health and Medicine University of Tasmania Burnie Tasmania Australia; ^4^ Ministry of Health and Medical Education Tehran Iran; ^5^ Department of Maternal and Child Health Nursing School of Nursing and Midwifery University of Cape Coast Cape Coast Ghana; ^6^ School of Nursing and Midwifery Edith Cowan University Joondalup Western Australia Australia; ^7^ School of Nursing and Midwifery Centre for Quality and Patient Safety Institute for Health Transformation Deakin University Geelong Victoria Australia

**Keywords:** barriers, facilitators, meta‐aggregation, postpartum care, qualitative systematic review

## Abstract

**Introduction:**

Postpartum care is crucial for maternal and infant health but remains inconsistently administered. This systematic qualitative review aimed to synthesize health care providers’ perspectives by exploring the barriers and facilitators experienced by health care providers in administering optimal maternal care during the first 12 months after childbirth.

**Methods:**

A systematic qualitative meta‐aggregation review was conducted using the Joanna Briggs Institute methodology. Peer‐reviewed qualitative studies from January 2010 to April 2024 were retrieved from MEDLINE, CINAHL, Embase, and Web of Science. Findings were mapped onto the Behavioral Model of Health Services Use.

**Results:**

Twenty‐three studies were included. Four key themes emerged: (1) professional barriers associated with administering quality health care, (2) organizational and systemic challenges in postpartum care (structural, policy, and resources barriers), (3) facility levels of services and their influence on decision‐making, and (4) client's factors impacting their postpartum. Facilitators included interprofessional teamwork, training, and continuity of care. Key facilitators that supported health care providers in administering postpartum maternal care included interprofessional teamwork, targeted training, and continuity of care.

**Discussion:**

Postpartum care continues to be fragmented and underprioritized at individual, organizational, and systemic levels, limiting the quality and continuity of maternal care. Addressing these challenges requires strengthened provider training, improved referral systems, culturally sensitive care, and integrated, team‐based approaches. Policy reforms that prioritize postpartum care as a core component of the maternal health care continuum are essential to improving maternal outcomes.

## INTRODUCTION

The postpartum period is a critical time for family health, emphasizing the need for effective care to support the physical and mental well‐being of individuals who are childbearing, newborns, and families.^1^ Maternal well‐being following childbirth plays a critical role in shaping the health outcomes of both women and their families over time.^2^ Poor practices in postpartum care can impair physical and emotional recovery, leading to lasting health effects.^3^ Furthermore, inadequate postpartum care can result in issues such as postpartum depression (PPD), anxiety, chronic pain, and even long‐term complications, such as pelvic floor dysfunction or cardiovascular disorders.^2^ To mitigate these risks, evidence‐based practices are essential to improve postpartum health care quality and reduce long‐term morbidity.^4^

**QUICK POINTS**

✦ Health care providers face professional, organizational, systemic, and client‐related barriers in administering postpartum care, including inadequate staffing, limited training, fragmented referral pathways, cultural barriers, and low maternal health literacy.
✦ Effective postpartum care is supported by interprofessional teamwork, provider training, continuity of care, family involvement, and culturally sensitive practices.
✦ Strengthening training, improving referral systems, promoting integrated, team‐based approaches, and prioritizing postpartum care in health care policy are essential to improve maternal outcomes.

Findings from a previously conducted systematic review identified a broad range of challenges and enablers that influenced women's engagement with health care professionals during the first year after childbirth.^5^ Normalizing women's postpartum health problems was a key challenge resulting in women not seeking professional help.^5^, ^6^ Other researchers highlighted a significant gap in the provision of postpartum care due to a lack of knowledge among health care providers (HCPs) regarding postpartum maternal morbidities.^7^, ^8^ Several other factors prevented women from seeking necessary postpartum help. These included knowledge deficits, low health literacy, fear of judgment, reliance on informal support networks, limited access to professional care and reliance on familial advice, and cultural influences.^5^ These challenges have compounded women's reported dissatisfaction with postpartum care compared with their more positive experiences during prenatal care.^9^

Beyond women's experiences, HCPs also face multiple system‐level challenges in administering optimal postpartum care. Studies have reported that limited staffing, time constraints, fragmented care models, lack of continuity, and insufficient training in managing postpartum complications hinder the ability of HCPs to provide comprehensive follow‐up and support.^10^ Moreover, institutional priorities often focus more on antenatal and intrapartum care, leaving the postpartum period underresourced and undervalued within health care systems.^11^ Understanding these barriers from the providers’ perspective is crucial to designing effective interventions and improving service provision.

## METHODS

A systematic qualitative meta‐aggregation review was conducted, following the Joanna Briggs Institute (JBI) process.^12^ Grounded in pragmatism, meta‐aggregation distills findings from qualitative studies into practical “lines of action” that can inform policy and practice.^13^ This approach systematically synthesizes evidence into actionable, evidence‐based statements while preserving participants’ perspectives. To ensure a comprehensive and methodologically rigorous synthesis, the review included an extensive search of relevant studies, minimizing bias and directly addressing the research question. The protocol for this review was registered with PROSPERO (registration number CRD42021266376).

### Conceptual Framework

The Behavioral Model of Health Services Use (BMHSU) was used as a framework for this study.^14^ The BMHSU (Figure 1) proposes that health outcomes are influenced by a combination of contextual and individual characteristics and health behaviors. For this review, contextual and individual characteristics were most relevant and further categorized into predisposing variables: enabling and need‐related variables.^14^ Contextual characteristics include family, society, and the health care system, whereas individual characteristics refer to personal beliefs about health care services, education level, and demographics such as age.^15^ Health behaviors, shaped by personal practices and the process of medical care, directly influence the use of personal health care services.^14^

**Figure 1:**
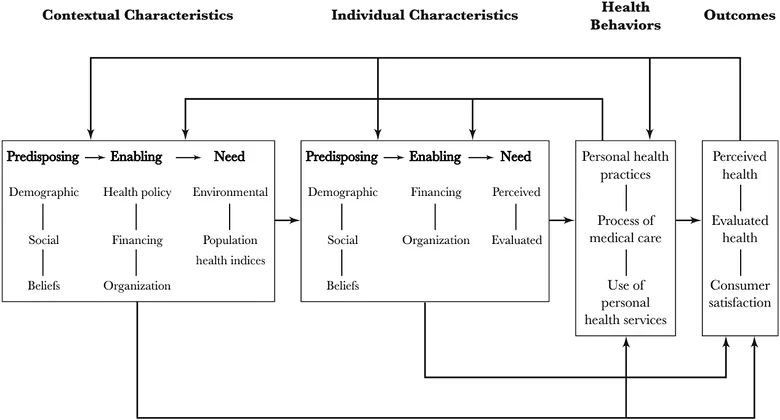
The Behavioral Model of Health Services Use Including Contextual and Individual Characteristics

Findings were deductively organized according to the BMHSU domains—predisposing, enabling, and need‐related factors^14^—providing a structured framework to interpret evidence and clarify how individual, organizational, and client‐level factors may interact to influence postpartum care.

### Search Strategy

This review included English‐language qualitative studies published between January 2010 and April 2024, sourced from MEDLINE (Ovid), CINAHL, Embase, and Web of Science. Qualitative methods capture the depth and context of HCP experiences, which quantitative approaches cannot. Including a broad publication range allowed identification of historical and contemporary trends, ensuring a comprehensive understanding of postpartum care provision by HCPs. The search strategy also intentionally included studies from diverse countries in order to capture a wide range of HCP experiences, organizational contexts, and cultural influences. Geographic diversity enhances transferability, identifies common and context‐specific barriers and facilitators, and strengthens the robustness and applicability of the findings for informing policy and practice.

The inclusion criteria were formulated according to Participants, Phenomenon of Interest, Context, and Outcome format adapted for qualitative research.^16^ For this review “Participant” was defined as all HCPs providing postpartum care, including general practitioners, midwives, maternal and child health nurses, obstetricians, and gynecologists in both private and public practice. “Interest” encompassed any factors hindering or influencing health care administration of PPC. “Context” included all international settings where postpartum care is provided. The primary “Outcome” was identification of barriers and facilitators to the provision of postpartum care (Supporting Information, Table S1).

Eligible studies containing qualitative data from any methodology or research design, including mixed‐methods studies with qualitative data as the primary data source, were considered for inclusion. All studies reporting quantitative data, including mixed‐methods studies with quantitative data as the primary data source, grey literature, abstracts, reviews, editorials, commentaries, and conference procedures, were excluded.

### Data Extraction and Quality Appraisal

Articles were selected based on rigorous inclusion and exclusion criteria. The following data were extracted: author(s), publication date, country, aims, methodology, method, phenomenon of interest, setting, participants, population ethnicity, reporting of morbidity, data analysis, and findings (Table 1).

**Table 1:** Summary of Included Studies in Chronological Order

Author (Year)	Aim	Methodology	Participants	Region	Recruitment	Data Collection	Data Analysis	Findings	Critical Appraisal
Lugina et al (2001)^17^	To explore midwives’ views in relation to the provision of systematic PPC	Grounded theory	N = 49 nurse/midwives	Dar es Salaam, Tanzania	Public health care facilities providing maternity and PPC services	Semistructured, in‐depth interviews	The constant comparative method	Eight categories were identified: “reflecting,” “getting ready,” “defining abilities,” “networking,” “integrating,” “balancing,” “dealing with reality,” and “caring.” The identified core category that integrated and encapsulated all other categories was “becoming a good resource and support person for postpartum woman.”	9/10
Forster et al (2006)^18^	To describe staffing and related issues in the provision of PNC in public hospitals in Victoria from the caregivers' perspective; and to explore what impact (if any) providers believe staffing issues have on their ability to provide high‐quality care to women	Mixed‐methods approach	N = 38 key informants: unit managers, associate unit managers, and clinical midwives	Victoria, Australia	Stage 1: A structured questionnaire was sent to all public hospitals in Victoria that provided PNC (n = 73), exploring the structure and organization of care (eg, staffing, routine observations, policy framework, and discharge planning). Stage 2: 14 maternity units were selected and invited to participate in a more in‐depth exploration of PNC.	Semistructured interviews	Thematic analysis of verbatim	There were significant issues associated with inadequate staff/patient ratios, staffing mix, patient mix, prioritization of birth suites over postnatal units, and the use of nonpermanent staff. Forty‐three percent of hospitals reported having only midwives (ie, no non‐midwives) providing PNC. Staffing issues impact hospitals’ ability to provide continuity of care.	8/10
Schmied et al (2008)^19^	To design and implement strategies to improve hospital‐based PNC at a Sydney metropolitan hospital	Qualitative descriptive study design	N = 31 midwives	Sydney, Australia	Health units of the Family Health Strategy	Open‐ended interviews	Thematic analysis of verbatim	Several important principles of PNC were described, including building a relationship with women, meeting their individual needs, being flexible in approach and providing continuity of care. “Listening to women,” “being there,” and “normalising experiences and expectations” were believed to be critical to achieving these principles.	9/10
Rayner et al (2008)^20^	To describe midwives’ views and experiences of the provision and organization of hospital‐based PNC	Mixed‐methods approach	N = 33 clinical midwives and midwifery managers	Victoria, Australia	Public hospitals	Semistructured interviews	Thematic analysis of verbatim	Midwives’ views and experiences of hospital‐based PNC are in accordance with women's views and experiences. They are less than satisfied with hospital‐based PNC, and they perceive a need for individualized, unrushed, flexible care.	8/10
Olsson et al (2011)^21^	To describe midwives’ reflections on counseling women at the postnatal check with special focus on sexual life after childbirth	Qualitative descriptive study	N = 32 midwives	Stockholm	Antenatal clinics	Focus group	Qualitative content analysis	Two themes emerged: balancing between personal perceptions of the woman's needs and the health care system restrictions and strategies for counseling about sexual life after childbirth. The midwives tried to create a picture for themselves of the woman coming for the postnatal check and her needs before the consultation. This picture guided the midwives, but lack of time and knowledge restricted them when counseling on sexual life after childbirth.	7/10
Brodribb et al (2013)^22^	To investigate the views and experiences of women and GPs about PPC in general practice	Qualitative descriptive study	N = 6 GPs	Queensland, Australia	Primary care services	Semistructured interviews	Content analysis	Three main themes emerged: the relationship between the woman and GP, practice management, and GP visits. This article focuses on the theme of GP visits and its subthemes: recommendations for GP visits, scope of practice, and content of a routine visit. Recommendations about GP visits given to women varied by birthing sector, obstetric provider, and model of maternity care, resulting in confusion among women about the timing and role of GPs in routine PPC. Similarly, GPs voiced concerns about a lack of consistent guidelines for their involvement in routine PPC. Although ideally placed to provide primary care to women and their infants in the postpartum period, the lack of consistent guidelines for the role of GPs is of concern to both the GPs and early parenting women.	9/10
Martin et al (2014)^23^	To explore important domains of women's postpartum experiences as perceived by women who are postpartum and obstetricians/midwives and to investigate how PPC could enhance patient preparation for the postpartum period	Qualitative descriptive study	N = 13 obstetric clinicians (2 midwives, 8 attending obstetricians, and 3 resident obstetricians)	USA	A large urban teaching hospital	Focus groups	Grounded theory	(1) Lack of women's knowledge about postpartum health and lack of preparation, (2) lack of continuity of care and absence of maternal care during the early postpartum period, (3) disconnect between providers and women who are postpartum, and (4) bridging the gap through suggestions for improvement	9/10
Rayner et al (2013)^24^	To gain a further understanding of this by exploring care providers’ views and experiences of PNC in private hospitals	Qualitative descriptive study	N = 11 HCPs	Victoria, Australia	PNC in private hospitals	Semistructured interviews	Thematic analysis	Two global themes were identified: “Constrained Care” and “Consumer Care.” “Constrained care” demonstrates the complexity of the provision of PNC and encompasses midwives’ feelings of frustration with the provision of PNC in a busy environment complicated by staffing difficulties, a lack of continuity, and the impact of key players in PNC (including visitors, management, and obstetricians). “Consumer care” describes care providers’ views that women often approach private PNC as a consumer, which can impact on their expectations and satisfaction with PNC. Despite these challenges, care providers, particularly midwives, highly valued (and generally enjoyed working in) PNC.	8/10
Santos Junior et al (2013)^25^	To report experiences of Brazilian physicians and nurses caring for women with PPD in primary health care settings	Qualitative descriptive study	N = 17 professionals (10 nurses and 7 physicians)	Campina Grande, Brazil	Health care units of the Family Health Strategy	Open‐ended interviews	Inductive content analysis	The major themes included limited professional exposure to PPD, PPD as the domain of psychiatry, and challenges in dealing with PPD. In the quotations, the term “n” refers to a nurse and “p” refers to a physician followed by the participant identity number.	9/10
Sialubanje et al (2014)^26^	To identify psychosocial and environmental factors contributing to low use of maternal health care services	Qualitative descriptive study	N = 176 community health care workers and nurse‐midwives	Kalomo District, Zambia	Five rural health centers	Focus group	Content analysis	Perspective on maternal health problems, health care–seeking behavior, and use of maternal health care services (ese of ANC service, use of birth services, use of PNC services). Personal and environmental factors contributing to poor use of maternal health care included services’ (negative) attitude toward HCPs and quality of maternal health care services, opinions of important others, and perceived and actual barriers.	7/10
Mselle et al (2017)^27^	To examine how PPC was administered in 3 postnatal hospital clinics in Dar es Salaam, Tanzania	Qualitative study	Ten nurse‐midwives and 3 obstetricians	Dar es Salaam, Tanzania	Face‐to‐face semistructured interviews	Referral regional hospital	Discourse analysis	Postpartum education was seen to be an urgent matter; there was a lack of supportive resources and infrastructure in the hospital clinics, and nurse‐midwives and obstetricians had to negotiate conflicting health and traditional discourses using various strategies. Nurse‐midwives and obstetricians are well positioned to provide life‐saving postpartum education; however, improvements are required, including increased number of nurse‐midwives and obstetricians.	8/10
Pallangyo et al (2017)^28^	To explore HCPs’ perceptions of the current PPC practice and its potential for improvement at governmental health care institutions in low‐resource suburbs in Dar es Salaam, Tanzania	Qualitative descriptive design	N = 53 registered, enrolled, and trained nurse‐midwives and medical and clinical officers	Dar es Salaam, Tanzania	Focus group	Health care institutions	Qualitative content analysis	The HCPs perceived that PPC was suboptimal and that they could have prevented maternal deaths. PPC was fragmented at understaffed institutions, lacked guidelines, and was organized in a top‐down structure of leadership. The participants called for improvement of organization of space, time, resources, communication, and referral system; providers’ knowledge; and supervision and feedback. Their motivation to enhance PPC quality was high.	8/10
Talbot et al (2018)^29^	To explore GPs’ views and experiences of using the postnatal check as a health‐related behavior change opportunity	Qualitative investigation	N = 18 qualified or trainee GPs	Northwest UK	Semistructured telephone interviews	General practices	Thematic analysis	The postnatal check is an unrealized opportunity for facilitating health‐related behavior change: opportunity for health‐related behavior change, role responsibility, and patient‐led vs GP‐led behavior change.	9/10
Lundeen et al (2019)^30^	To understand the experiences of providers of group ANC/PNC and compare their job satisfaction and perceived stress with individual ANC/PNC providers	Mixed‐methods study	N = 20 provider participants in each group	Rwanda	Focus groups and questionnaires	Five health centers	Thematic analysis	Obstacles to women's access: costs, distance, poor attendance	7/10
Alnuaimi et al (2020)^31^	To explore Jordanian health care professionals’ perspectives about sexual education after giving birth	Descriptive qualitative approach	N = 22 midwives, nurses, and obstetricians	Jordan	Primary health centers	Semistructured, in‐depth focus groups	A manual thematic content analysis tool	Five major themes emerged: silence, resumption of sexuality after giving birth/area of conflict, men's authority in resumption of sexuality, the importance of sexual education (what, when, and whom), and suggestions for sexual education approaches.	7/10
Hyzam et al (2020)^32^	To examine the health education and promotion patterns, health‐seeking behavior of women, and barriers to the use of maternal health care services from public health care facilities	Qualitative study design	N = 9 health care leaders and midwives	Rural areas of Yemen	Public health care facilities	In‐depth interviews and focus group discussions	An inductive and deductive thematic content analysis	Ad hoc and parallel health education and promotion activities, poor coverage of health promotion programs, policy‐related or system‐related factors, a woman confirmed part of the narrative of the midwife, and provider‐related factors	8/10
Verma and Mukherjee (2020)^33^	To examine the barriers perceived by nurses in maternal and newborn care at community health care centers in rural areas of India	Qualitative phenomenology	N = 6 nurses based on their experience in maternal and newborn care	Madhya Pradesh, India	Community health centers	In‐depth interviews of nurses	Qualitative data analysis	Barriers observed by nurses: (1) manpower linked (shortage of nurses, shortage of specialist physician, shortage of supporting staff), (2) facility linked (infrastructure, lack of medicine, inadequate basic facilities inventory management, security and safety threats), (3) nurses linked (unclear task shift to nurses, no promotion opportunity), and (4) culture linked	8/10
Weldearegay et al (2020)^34^	To assess the quality of and barriers to routine childbirth care signal functions during the intrapartum and immediate postpartum period	A sequential explanatory mixed‐methods study	N = 225 skilled birth attendants	Tigray, Ethiopia	Semistructured questionnaires and focus group discussions	The primary‐level health care facilities	Content analysis	Poor quality of care during birth and immediate postpartum related to work‐related burnout, gap between providers’ skill and knowledge, lack of enabling working environment, poor motivation scheme and issues related to retention, poor providers’ caring behavior, inability to translate training into practice, mismatch between number of provider and facility client flow for birth, and in availability of essential medicine and supplies	7/10
Poon et al (2021)^35^	To explore the experiences of PCPs in managing women who are postpartum	Qualitative research method	N = 29 GPs	Singapore	Focused group discussions and in‐depth interviews	Private practice and various public primary care clinics	Content analysis	PCPs’ personal attributes, such as gender and knowledge, influenced their PPC provision. Prior training, child caring experience, and access to resource materials contributed to their information mastery of PPC. Their professional relationship with local multiethic and multilingual Asian women was impacted by their mutual communication, language compatibility, and understanding of local confinement practices. Consultation time constraint, awareness of community postnatal services, and inadequate handover of care from the specialists hindered PCPs in the health care system.	8/10
Ruderman et al (2021)^10^	To understand providers’ perspectives on challenges in PPC to highlight strategies for optimizing care	Qualitative descriptive study	Chicago, IL, USA	N = 26 HCPs	Northwestern Memorial Hospital's Prentice Ambulatory Care Clinic	Focus groups were conducted using a semistructured interview	Constant comparative method	Challenges providers perceived for patients and those they perceived for themselves often overlapped, including difficulty with appointment scheduling, insurance limitations, lack of provider continuity, and knowledge gaps. Providers identified ease of referrals to specialists, access to tangible services (eg, contraception), and enhanced care coordination (eg, patient navigation) as potential facilitators of improved PPC.	8/10
Pope et al (2023)^36^	To examine HCPs’ experiences and attitudes regarding maternal psychological well‐being, and the provision of perinatal mental health support, including during the COVID‐19 pandemic	Qualitative study	Ireland	N = 18 health care professionals	Clinical practices	Semistructured interviews	Inductive thematic analysis	Four themes were developed: “Supporting women in health care settings,” “Skills and capacity to provide adequate care,” “Structural barriers to care provision,” and “The impact of the COVID‐19 pandemic on stress support.”	8/10
Bains et al (2023)^23^	To identify health care professionals’ views on the current accessibility and acceptability of perinatal mental health services and ways of improving services by addressing the barriers for these women	Qualitative research	24 health care professionals	UK	Nationwide HCPs	Semistructured interviews	Ritchie and Spencer's framework method analysis	Three main themes were identified from the data: (1) lack of awareness and understanding of perinatal mental illness and service structure in both health care professionals and patients; (2) patients’ relationships with family, friends, and health care professionals can both hinder and facilitate access to services; (3) health care professionals encourage raising awareness, flexibility, developing shared understandings, and questioning assumptions to improve the accessibility and acceptability of services.	8/10
Akongo et al (2024)^37^	To explore the barriers and facilitators to accessing PPD health care among those affected	Mixed‐methods study	Six key informants	Uganda	Regional referral hospital	A facility‐based survey and focus groups and interviews	Content analysis	Key barriers to seeking formal care included stigma and lack of social support, low income levels, and availability of other health care options such as the presence of herbalists. Perceived psychological distress, health education, linkage, and psychological support to the women facilitated care seeking.	7/10

**Abbreviations**: ANC, antenatal care; GP, general practitioner; HCP, health care provider; PCP, primary care provider; PNC, postnatal care; PPC, postpartum care; PPD, postpartum depression.

All articles retrieved from the databases were imported into Covidence^38^ with duplicates removed prior to selection of studies (Figure 2). Screening was conducted in 2 stages: first by title and abstract, followed by a full‐text review. Two reviewers (M.R., T.D.A.) independently assessed the full text of eligible articles using the JBI Critical Appraisal Checklist for Qualitative Research.^39^ This checklist consists of 10 questions organized into 3 main sections: filtering, technical appraisal, and theoretical appraisal. Studies were considered for inclusion if both reviewers answered “yes” to at least 7 of the 10 questions. Any discrepancies were resolved through discussion and consensus after a thorough review of the checklist criteria.

**Figure 2:**
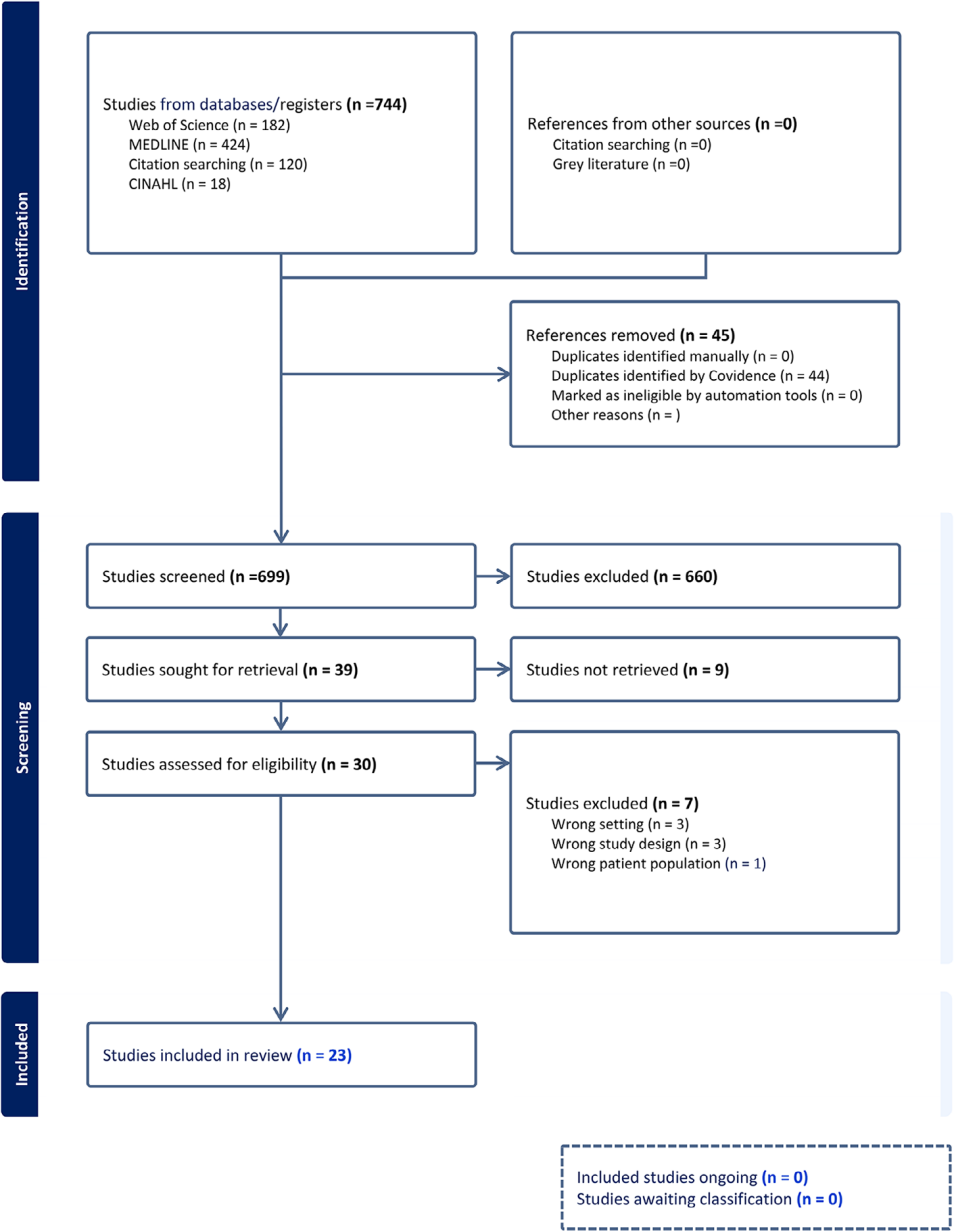
Flow Diagram of Trial

In addition to the critical appraisal, qualitative findings relevant to postpartum care provision were independently extracted by the same 2 reviewers using a standardized data extraction form. Extracted data included participant quotations and interpreted themes directly related to barriers and facilitators. Discrepancies in data extraction were resolved through discussion and consensus, and all extracted data were cross‐checked to ensure accuracy and consistency prior to synthesis. Critical appraisal tools were applied to assess the trustworthiness, relevance, and rigor of included studies. The credibility of qualitative research was emphasized, and high‐quality articles without apparent bias were prioritized.^40^ Multiple interpretations of qualitative findings were considered to achieve a comprehensive understanding of the aims of the included studies.

### Data Synthesis

Meta‐aggregation was used to synthesize data and generate a set of statements that represent the findings. It provides a structured, transparent, and practical method to synthesize qualitative evidence across studies, turning diverse findings into clear, actionable insights that can directly address the research question about HCP experiences and system‐level factors. These were then categorized based on similarity in meaning. These categories were synthesized to produce a single comprehensive set of findings that can be used to inform decision‐making and evidence‐based practice.

## RESULTS

The initial search strategy yielded 972 articles, with 23 articles included in the review (Figure 2). Barriers and facilitators to HCPs’ provision of postpartum care were collated and interpreted using the JBI Qualitative Assessment and Review Instrument (JBI‐QARI) within the BMHSU framework.^14^ This framework categorizes factors influencing postpartum care into 3 domains: (1) predisposing factors, encompassing institutional conditions that may encourage or limit HCPs’ service provision regardless of women's use; (2) enabling factors, including conditions that support or hinder HCPs in providing timely and effective care; and (3) need‐related factors, representing recognized health needs or conditions identified by HCPs and women.

Twelve categories of barriers and facilitators were extracted from the included studies and aggregated into 4 synthesis statements (Table 2). The importance of each category was determined based on the number of supporting studies and the methodological rigor assessed via JBI‐QARI, with categories supported by greater evidence prioritized in the synthesis. This approach provides a transparent and structured interpretation of key contextual factors influencing access to postpartum maternal care and highlights the central role of HCPs in ensuring care accessibility and quality.

**Table 2:** Meta‐Aggregation of Barriers and Facilitators to HCPs’ Provision of Maternal Care in the First 12 Months Postpartum

Synthesized Findings	Category	Included Studies	Findings
Synthesis statement 1: professional barriers associated with providing quality health care HCPs face challenges in postpartum care because of insufficient staffing, limited consultation time, lack of mental health training, and gender‐related biases. These barriers affect the quality of care and communication.	Inadequate staffing levels and support resources	^20^, ^24^, ^28^, ^30^, ^31^, ^32^, ^33^, ^34^, ^36^	N = 18
	Limited knowledge and skills for providing mental health support	^18^, ^24^, ^25^, ^29^, ^34^, ^36^, ^37^	N = 12
	Compromised care because of time constraints	^21^, ^22^, ^29^, ^31^, ^35^, ^36^, ^37^	N = 8
Gender‐specific challenges	^32^, ^35^	N = 2
Synthesis statement 2: organizational and systemic challenges in postpartum care—structural, policy, and resources barriers Organizational and systemic challenges in postpartum care stem from limited resources, inadequate policy frameworks, high service costs, and sociocultural barriers. These factors impede access to quality care, delay referrals, and contribute to inefficiencies in service provision, ultimately affecting the overall care experience for new mothers.	Lack of clear referral pathways	^31^, ^36^	N = 7
	Operational costs of services	^10^, ^17^, ^32^	N = 3
Social and cultural barriers	^10^, ^17^, ^19^, ^31^, ^36^, ^41^	N = 8
	Health care professional‐patient dyad	^23^, ^36^	N = 4
Synthesis statement 3: facility levels and their influence on decision‐making Facility levels greatly influence postpartum care decision‐making. Lower‐level facilities face resource and training limitations, hindering effective care, whereas higher‐level facilities, despite better resources, are affected by policy and cultural barriers. These factors shape the quality and consistency of postpartum care.	Limited training opportunities and support for HCPs	^10^, ^36^, ^37^	N = 9
	Care provision model incorporating family participation	^19^, ^23^, ^24^, ^31^	N = 9
Synthesis statement 4: client's factors impacting their postpartum Low health literacy and limited access to services can hinder understanding and timely postpartum care, increasing health risks. Cultural practices and reliance on traditional healers may delay medical treatment, creating gaps in essential care.	Health literacy gap and service access barriers	^10^, ^26^, ^27^, ^37^, ^41^	N = 9
	Cultural Health practices and use of traditional healing method	^37^	N = 4

Abbreviation: HCP, health care provider.

### Synthesis Statement 1. Professional Barriers Associated With Providing Quality Health Care

#### Category 1: Inadequate Staffing Levels and Support Resources

Inadequate staffing and resourcing were frequently cited as a major obstacle to providing comprehensive postpartum care for women in 11 out of 23 studies.^18^, ^20^, ^24^, ^28^, ^30^, ^31^, ^32^, ^33^, ^34^, ^36^, ^42^ For example, in Pallangyo et al,^28^ one HCP stated, *“*We will not be enough to provide proper PPC. We are already overwhelmed so we suggest also for the increased number of HCP and especially specifically assigned to the role.”^28^

Additionally, a shortage of specialist doctors was reported as a barrier to providing postpartum care: “There are many challenges that are preventing us [healthcare providers] from providing basic and essential health services to pregnant women. We don't have adequate female doctors and midwives, drugs.”^32^

Living in rural areas and staff shortage were also reported as barriers to the provision of postpartum care and supported in one study: “Recruitment is usually an ongoing issue due to the hospital being in a rural area.”^34^

Two studies reported the lack of diagnostic facilities as a barrier to providing postpartum care.^33^, ^34^ For example, “There is lack of investigation services [diagnostic] on twenty‐four‐hour basis.”^33^

A lack of financial support for education and limited access to professional development opportunities due to various factors, such as poor internet connectivity in low‐resource settings, were reported as barriers to professional growth and career advancement: “After completion of course our skills improve but financial raise [funding?] should be there it will sustain our motivation.”^33^

HCPs reported that excessive clerical tasks, such as paperwork, overwhelmed them and reduced time for direct woman care.^19^

The results from Alnuaimi et al^31^ and Pope et al^36^ highlighted key challenges in maternal mental health care, including insufficient organizational investment and resources, such as inadequate referral pathways, long wait lists, and insufficient public services tailored to women’ needs, leaving them without prompt access to adequate care.

#### Category 2: Limited Knowledge and Skills for Providing Mental Health Support

Eight studies reported that HCPs lacked adequate knowledge regarding mental health disorders and treatments. Here, one provider admitted, “Here in our community, we don't have any cases of postpartum depression. I mean, either we don't have any or we don't know how to observe [diagnose it].”^25^

Mental health disorders were often misunderstood or considered normal, delaying diagnosis and care.^36^ Reliance on laboratory tests rather than clinical judgment was common: “HCPs relying on laboratory results to assess and confirm the presence of PPD.”^37^

One study highlighted that breastfeeding support often overshadowed mental health care: “The biggest, biggest, biggest thing is always the breastfeeding.”^20^

Eight studies highlighted a lack of knowledge regarding mental health disorders and effective support and treatment options among HCPs.^17^, ^18^, ^24^, ^25^, ^29^, ^34^, ^36^, ^37^

#### Category 3: Compromised Care Because of Time Constraints

Short consultation times were a widespread problem. HCPs described their workload and limited time per patient.^21^, ^22^, ^29^, ^31^, ^35^, ^36^, ^37^ One participant mentioned:

While we would love to handle every patient well, this is not feasible because of time constraints. Here we see very many mothers and when you think of giving each one enough time to talk, the numbers pile up.^37^

Fragmented, limited, and hard‐to‐access postpartum support services led to unmet needs and feelings of isolation among women, with some HCPs explicitly admitting they “pass[ed] the buck” rather than taking ownership of care coordination.^36^

Time pressures also affected the ability of HCPs to address complex postpartum issues such as perineal tears.^21^

#### Category 4: Gender‐Specific Challenges

Gender preferences influenced postpartum consultations, with women favoring female HCPs for intimate examinations. One participant noted, “Some of them, they prefer a female doctor instead of a male doctor, especially if it involves a lot of checking (of) the breast and also the private part(s).”^35^

### Synthesis Statement 2. Organizational and Systemic Challenges in Postpartum Care: Structural, Policy, and Resources Barriers

Significant organizational and systemic challenges affecting the accessibility and quality of postpartum care were identified and supported by 22 findings. These challenges were grouped into 4 key categories: unclear referral pathways, high operational costs, social and cultural barriers, and issues within the health care professional‐patient relationship.

#### Category 5: Lack of Clear Referral Pathways

Seven studies highlighted a persistent gap in health care systems regarding the availability and clear referral pathways, particularly for mental health and psychosocial support.^21^, ^22^, ^29^, ^31^, ^35^, ^36^, ^37^ HCPs often identified women's needs but lacked clear and accessible pathway for timely referrals: “[W]e're telling these patients all the time… ‘talk to your GP,’… If they have taken those steps, …we have to have… people to refer them on to.”^36^

HCPs reported that women felt “lost in the system” because of inadequate referrals, leading to sentiments of being a “cog in a wheel” or HCPs “passing the buck.” Consequently, delaying referrals was viewed as a means to protect women from complex care systems and foster trusting relationships.^36^

#### Category 6: Operational Costs of Services

High out‐of‐pocket expenses associated with health care services in many facilities deterred women from attending follow‐up visits or seeking support^10^, ^17^, ^32^: “The cost of some health care services in the facilities prevent mothers from visiting the reproductive healthcare centres frequently.”^32^

#### Category 7: Social and Cultural Barriers

Social and cultural factors further limited access to postpartum care. Language barriers and low socioeconomic status hindered effective communication between women and HCPs, especially when English was a second language and interpreter services were unavailable.^36^ Cultural values, taboos, and the fear of judgment often prevented women from discussing postpartum issues, such as breastfeeding, hygiene, or sexual health^17^, ^31^, ^41^:

Sometimes our culture and norms inhibit the communication between our clients and us as they considered opening this issue is a shame and forbidden…so I think if we start the chat, women will accept the discussion and be encouraged to ask questions.^31^

Feelings of shame and distrust contributed to social isolation, depriving women of needed postpartum support.^10^, ^19^, ^36^ To address this, some HCPs emphasized strategies such “listening,” “clarifying expectations,” “providing relevant information,” and “facilitating a restful environment” to support the women's emotional and practical needs.^19^ One provider emphasized, “The biggest skill that I feel I bring to a mother as…a health care professional, is my ability to listen, and to acknowledge her feelings.”^36^

#### Category 8: Health Care Professional‐Patient Dyad

The quality of the health care professional‐patient relationship played a crucial role in shaping postpartum care experiences, particularly in relation to continuity of care. One HCP noted, “Patients who have been [seen] by the same provider and they've been lucky enough to get that continuity… that's an increased likelihood that they're going to return.”^10^

Strong, continuous relationships between providers and postpartum women encouraged active participation in care and improved outcomes.^36^ Another HCP stated, “Patients who have been [seen] by the same provider and they've been lucky enough to get that continuity …, that's an increased likelihood that they're going to return.”^10^

However, divergent perspectives on expected care outcomes occasionally led to miscommunication:

…if you investigate this problem [urinary incontinence] there's a limited utility to it. You're not going to do a lot of things to fix this problem at this point. So, you're just asking a question that's going to lead you to a place that you can't do any help for.^23^

Such differences could discourage women from seeking help or following care plans, affecting recovery.

### Synthesis Statement 3: Facility Levels and Their Influence on Decision‐Making Processes

#### Category 9: Limited Training Opportunities and Support for HCPs

Midwives and other HCPs faced substantial challenges in providing comprehensive postpartum care, especially for PPD and other mental health issues, because of insufficient formal education and training. Many providers lack the necessary skills to effectively use postpartum mental health screening tools, limiting early identification and intervention.^36^ The role of hospital management is crucial in prioritizing postnatal care, but, without robust institutional backing, providers struggled to implement best practices when caring for women who were postpartum.^24^, ^28^

Although breastfeeding education remained a key priority, it was often overshadowed by other important aspects of postnatal care, including mental health and relationship support^17^, ^20^:

[Y]ou'd be learning about…postpartum depression and puerperal psychosis and things that are as rare as hen's teeth…[but] when you come out into the real world, you realise: …that isn't the reality of most of the problems that people face…it's all the normal stuff.

#### Category 10: Care Provision Model Incorporating Family Participation

Family involvement in postpartum care was recognized as beneficial. Providing written educational materials to women and their families supports learning and empowers them to manage care at home.^31^ These resources can be crucial in reinforcing key information about infant and maternal health. One provider explained, “I think offering written materials about sexuality would be a good idea, then they can discuss the issue with their husbands at home.”^31^ Inclusive postpartum education sessions helped equip family members to support both woman and infant, enhancing continuity of care.^27^

Current postnatal care systems often focus narrowly on women with prenatal complications, excluding many women who are postpartum and would benefit from broader support.^24^

In general, there is no physical examination for mothers after delivery at the reproductive health care centres. We [midwives] just provide blood sugar test for mothers who need sub‐dermal contraceptive. Mothers come after delivery if they feel tired, her baby is sick, for vaccination, and family planning services.^32^

This lack of comprehensive follow‐up highlights gaps in postpartum monitoring.

Home visits have been identified as an effective intervention, particularly for women experiencing PPD, enabling early detection and support^25^: “Maybe we could make more groups, more home visits, listen to the complaints. For the disease [postpartum depression], we don't offer anything here besides the referral.”^25^

Continuity of care with a familiar provider is crucial but often lacking, leading to gaps in support.^19^, ^23^, ^24^ One HCP noted,

…if the patient knows you and feels comfortable with you, they're just gonna talk to you and say “Hey this is bugging me” or whatever. But if the patient doesn't know you, she's just kinda… sitting there, waiting for you to ask…^23^

Establishing dedicated counseling clinics for women to provide ongoing advice, consultation, and mental health support ensured comprehensive care for everyone, regardless of postpartum complications^31^: “Consultation for sexuality in a private office would be a good idea to help all clients especially for people who feel embarrassed to share such subject.”^31^

### Synthesis Statement 4: Client Factors and the Impact on Postpartum Care

#### Category 11: Health Literacy Gap and Service Access Barriers

Low health literacy among women who are postpartum, defined as limited capacity to obtain, process, and understand basic health care information and services needed to make appropriate health decisions, significantly hindered HCPs’ capacity to follow up on postpartum complications.^10^ Women with low health literacy were less likely to access antenatal care, which increased the likelihood of home births without prior medical booking. Births occurring at home, where women had limited or no engagement with formal prenatal services, left them particularly vulnerable to poor postnatal outcomes.^22^, ^26^ Improving postpartum care accessibility and acceptability therefore requires raising awareness about available services, fostering flexibility in service provision, and promoting shared understanding between women and providers.^41^ Family support was also found to be limited, especially for women experiencing mental health issues, reflecting a broader health literacy gap within families, wherein family members’ limited understanding of health needs restricted their capacity to support women who were postpartum effectively. One participant noted:

Just even knowing that the services exist, or you know, the way in which mental health is spoken about within the home, or is recognized in itself, probably places a barrier, in terms of those women accessing support, knowing when they need to access support, and knowing when things are becoming, you know, unsafe, or becoming a problem.^41^

Additional barriers included limited childcare availability and lack of transportation, which prevented many women from attending postpartum appointments. As one HCP explained, “The most common reasons that I hear are I didn't have a way to get to the clinic or I don't have anyone to watch my kids.”^10^

Transportation issues were echoed in other studies, wherein missed postpartum reviews were attributed mainly to “lack of transport.”^26^

#### Category 12: Cultural Health Practices and Use of Traditional Healing Method

Akongo et al^37^ reported that women often sought care from traditional healers and herbalists within their communities:

Where these mothers deliver from affects their health‐seeking behavior. Because those who deliver from home and experience mental health distress symptoms, may believe that these symptoms are a nonbiomedical illness, they seek care from traditional healers or any other place of their choice.^37^

Women sought help only when psychological distress became severe, partly because of the lack of formal linkages between home births and health care facilities. Confusion surrounding the symptoms of PPD often resulted in inappropriate care choices.^37^

## DISCUSSION

The study highlights critical barriers and facilitators that HCPs encounter when providing postpartum care, offering valuable insights into areas for improvement and intervention that can be further understood through the BMHSU domains of enabling, predisposing, and need‐related factors. These results demonstrate that some challenges are rooted in systemic issues, whereas others reflect professional limitations and societal biases, emphasizing when targeted support could improve care provision.

### Enabling Factors

One of the most prominent barriers identified was inadequate staffing and resources, an enabling factor within the BMHSU. HCPs frequently reported feeling overwhelmed by patient volume and role demands, which constrained their capacity to provide comprehensive, individualized care during the postpartum period. Although underresourcing in health care is widely recognized,^43^ insufficient allocation of resources—specifically in postpartum care—critically affects both the quality and timeliness of interventions provided to women.

Time constraints also emerged as a recurring challenge. Increasing patient loads and limited consultation times prevented many HCPs from engaging meaningfully with patients, thereby affecting the quality of care.^44^, ^45^, ^46^ This was particularly concerning in the postpartum period, when women required additional attention to address their emotional, physical, and practical recovery concerns.

### Predisposing Factors

Another significant barrier was the lack of specialized knowledge and skills in managing specific postpartum conditions, particularly mental health issues such as PPD.^47^ This gap highlights the need for targeted education and professional development to ensure HCPs are equipped to address the complex needs of women who are postpartum. Samuel et al^46^ also noted that factors such as cost, time, and perceived relevance influenced HCPs’ capacity and capability to undertake further training.

A more nuanced predisposing factor was gender‐related preferences. Many women requested female HCPs for intimate consultations related to breastfeeding, vaginal health, and mental health. When these preferences could not be met because of staffing constraints, it affected patient comfort and engagement in care. Although not addressed in the included studies, similar challenges may also arise for transgender and gender‐diverse individuals seeking postpartum care, wherein provider gender, inclusivity, and culturally safe communication may influence care engagement and experiences.

Despite these challenges, there were several facilitators that HCPs identified as contributing to more effective postpartum care. Strong teamwork among HCPs was frequently cited as a key factor in overcoming barriers to care. This is consistent with Selix et al,^48^ who suggested that the complexity of maternal care requires good teamwork and effective communication to formulate safe care plans. Collaborative efforts between obstetrician‐gynecologists, midwives, mental health professionals, and general practitioners allowed for more holistic care plans, ensuring that both the physical and emotional needs of women were addressed.^49^

### Need‐Related Factors

The need‐related domain represents the actual or perceived health needs that prompt postpartum care. HCPs reported that women often present with complex physical, emotional, and psychosocial needs, such as PPD, anxiety, perineal trauma, breastfeeding challenges, or complications after home births.^5^ When these needs were unrecognized or delayed, women experienced gaps in care and delayed interventions.^21^, ^25^, ^27^, ^37^ Additionally, cultural practices, including reliance on traditional healers, sometimes led to inappropriate or delayed care, highlighting the importance of timely assessment and culturally sensitive engagement by HCPs to ensure women's needs are addressed.

### Organizational and Systemic Barriers

Organizational and systemic challenges significantly influenced care quality and decision‐making in the current study. These included limited resources, gaps in policies addressing the unique needs of women who are postpartum, and a lack of coordinated referral pathways. Such barriers are common across health care services, with ongoing efforts to mitigate risks and improve patient outcomes.^50^ Our findings suggest that these barriers may contribute to poorer outcomes for women, particularly those from culturally diverse backgrounds, with low health literacy, or living in rural and remote areas.

Fragmented care, delays, and missed follow‐up appointments often resulted from poorly defined referral pathways, disproportionately affecting women with complex health needs.^51^ Operational cost constraints further limit access to essential services, prioritizing acute care over preventive postpartum support such as home visits and specialist services.^52^ Social and cultural barriers, including stigma, language difficulties, and distrust in health care systems, exacerbate disparities in care.^53^

Finally, the health care professional‐patient dyad remains central to effective care but is often strained by time pressures, insufficient systemic support, and lack of service integration.^45^ Expecting individual providers to meet the multidimensional needs of women who are postpartum without multidisciplinary or coordinated support places an unrealistic burden on HCPs and highlights the need for systemic interventions.

Themes of professional challenges, organizational and systemic issues, and client‐level barriers exist across all settings; however, the intensity and nature differ. Higher‐resourced countries report more professional and continuity‐related challenges, whereas lower‐resourced countries are dominated by structural, financial, cultural, and health literacy barriers.^54^ Thus, policy priorities and implementation strategies need to be tailored to the specific health care system context, ensuring that interventions address the dominant barriers within each setting.

### Implications for Policy and Practice

To strengthen postpartum maternal care, policy and service reforms are required that position the postpartum period as a core component of the maternity care continuum rather than an optional extension of care. National and institutional policies should establish clear, standardized frameworks that define the roles and responsibilities of HCPs—including midwives, general practitioners, nurses, and obstetrician‐gynecologists—in providing ongoing maternal follow‐up during the first 12 months after childbirth. Such frameworks should support coordinated, multidisciplinary care and promote continuity across maternity and primary health care services.

Health systems should also invest in structured postpartum care pathways that include routine follow‐up, early identification of physical and mental health complications, and timely referral mechanisms. Strengthening access to mental health resources, particularly screening and management of postpartum mental health conditions, is essential to ensure that HCPs can respond effectively to women's needs during this period.

In addition, workforce capacity must be supported through targeted training and professional development programs that equip HCPs with the knowledge and skills required to identify and manage postpartum morbidities. Addressing systemic barriers, such as staff shortages, limited consultation time, and unclear referral pathways, will be critical to enabling providers to provide consistent, evidence‐based postpartum care.

Finally, strengthening institutional support and policy accountability mechanisms can help reduce variability in postpartum service provision and improve the accessibility, quality, and continuity of maternal care across diverse health care settings and populations.

### Strengths and Limitations

This review has several notable strengths. To our knowledge, it is the first systematic qualitative meta‐aggregation examining the barriers and facilitators experienced by HCPs providing maternal care during the first 12 months postpartum. The use of meta‐aggregation, aligned with the JBI methodology, enabled the synthesis of high‐quality qualitative evidence while preserving the original meaning of participants’ experiences. In addition, a comprehensive and transparent search strategy across multiple databases ensured broad coverage of the literature. The application of standardized critical appraisal tools further enhanced the methodological rigor and credibility of the review. Importantly, the synthesis generated practice‐oriented insights by identifying actionable themes that can inform policy development, workforce support, and health care system improvements aimed at strengthening postpartum care.

Despite these strengths, several limitations should be acknowledged. First, restricting the review to English‐language studies may have excluded relevant evidence and introduced language bias. Second, the inclusion of only peer‐reviewed literature may have resulted in the omission of valuable grey literature, such as government reports, policy documents, and conference proceedings, which could provide important contextual insights. Third, heterogeneity across study designs, participant populations, and health care settings posed challenges for synthesis and may limit the generalizability of the findings. Finally, many included studies relied on self‐reported data, which may be subject to recall bias or social desirability bias, potentially affecting the validity and reliability of the findings.

## CONCLUSION

This systematic qualitative meta‐aggregation review highlights the complex interplay of barriers and facilitators influencing HCPs’ ability to provide maternal care during the first 12 months after childbirth. Organizational, systemic, and relational factors significantly shape care experiences and outcomes in the postpartum period. Addressing these challenges through clearer referral pathways, culturally responsive practices, adequate resourcing, and stronger structural support for HCPs is, therefore, essential for improving postpartum care. By synthesizing HCPs’ experiences across multiple studies, this review offers actionable insights for policy, workforce planning, and health care system redesign. Grounded in a pragmatic approach, qualitative meta‐aggregation preserves the context of individual studies while generating practical, evidence‐based recommendations. Therefore, this systematic review aims to identify the barriers and facilitators experienced by HCPs in providing optimal maternal care during the first 12 months after childbirth. Ultimately, the findings of this review provide a roadmap for targeted interventions that can enhance the quality, accessibility, and effectiveness of postpartum maternal care.

## CONFLICTS OF INTEREST

The authors declare no conflicts of interest.

## Supporting information




**Table S1**. Search Strategy Components

**Data Synthesis**. Barriers and Facilitators to Providing Post‐Childbirth Maternal Care by Health Care Providers in the First 12 Months After Childbirth: A Systematic Qualitative Meta‐Aggregation Review
