# Supplementary material for: Barriers and Facilitators to Providing Women Postpartum Care by Health Care Providers in the First 12 Months After Childbirth: A Systematic Qualitative Meta‐Aggregation Review
Source: J Midwifery Womens Health. 2026 Jun 16;71(5):820–40. doi: 10.1111/jmwh.70142 (PMC13655192; doi:10.1111/jmwh.70142)
Supplement: Supplementary file 1 [file JMWH-71-820-s001.docx]

Data synthesis

Barriers and facilitators to providing post-childbirth maternal care by health care providers in the first twelvemonths after childbirth: A systematic qualitative meta-aggregation review.

| **Author** | **Finding** | **Illustration** |
| --- | --- | --- |
| (Lundeen et al., 2019) | **Implementation challenges at the facility level and solutions**  The most commonly cited problem is **a shortage of provider staff at health centres**. When there are few staff available to cover all the basic service units at the health center, it is common for the health center director to allocate a single provider to several services at once —such as maternity, ANC, and family planning. Stress and chaos can result:  Peers in the FGD suggested that the health center should plan for just “one group in the morning . . . [and] another in the afternoon and you will manage to serve them all correctly and satisfactorily” (FG2 R5). In order to achieve this, providers encouraged one another to be proactive and advocate for a work schedule that allows for group ANC on most days of the week.  Solutions to these challenges suggested by providers during the FGDs include: | It happens sometimes for a service provider to feel stressed due to the fact that we work in more than one service; we get a challenge of failing to render an adequate service because the group care activities require you sit in one place--to stay there. It happens that you may be urgently needed in a different service; you are thus obliged to excuse yourself for an absence of little while to provide the services called for, and this becomes disturbance. You may be working from maternity and find yourself at the same time in post natal group care of those very mothers; so you feel puzzled. It is due to the little number of workers whereby we are bound to combine services because they are more than the number of workers available. (FG3 R6) p. 11  I have registers where I plan my work throughout the entire year. I present my plan every month so that the management will know where I will be working from. Yes of course all may depend upon the health center; but when I have presented my plan of activities, they elaborate a timetable taking into account that plan of mine. For example, they know that from Tuesday to Friday I work with group care for antenatal consultation services. (FG1 R9) p.11  Organize women who live close to the health center into groups that meet during the morning and women who live far from the health center into groups that meet in the afternoon (FG3 R1); • Cooperate with the insurance payment office so they will allow women to pay after the visit rather than require payment before the group visit begins (FG3 R6, FG2 R1); and Plan for group PNC on the same days women present for newborn immunizations (FG2 R10). P.12 |
| (Sialubanje et al., 2014) | **Perspective on maternal health problems**  Most respondents mentioned that many women were aware of the physical complications during pregnancy and childbirth and described various  complications ranging from minor abdominal pain and spotting during pregnancy to life-threatening complications including retained placenta, excessive bleeding, cord prolapse, hypertensive disorders and obstructed labour.  Pregnancy and childbirth complications were seen as resulting from poor maternal healthcare seeking behaviour, specifically poor antenatal care attendance and giving birth at home: Most women who give birth at home are the ones who face problems during labour because most TBAs and old women do not have enough knowledge to assist them (25-year-old nurse-midwife).  **Utilization of postnatal care services**  Most respondents mentioned that many women do not receive any postnatal care services from the clinic. They explained that most women only go to the clinic to take their children for under five clinics outside the postnatal period.  **Personal and environmental factors contributing to poor use of maternal healthcare services (Negative) attitude towards healthcare providers and quality of maternal healthcare services**  On the contrary, most nurses explained that staffing levels were low in the health centres, and that if nurses went out for workshops or ordering of medical supplies at the district medical office, health centres would be left unattended. They explained that in most cases, people in the community would know when the nurse was away from the health centre, and this made some mothers opt to deliver at home.  Concerning physical barriers, respondents stated that long distances to the health centres, poor road network and non-availability of transport prevented many women from completing antenatal care visit schedules and giving birth at the clinic.  In addition, participants mentioned that some women gave birth at home because of high transport costs and other indirect costs such as baby clothes and other requirements, and food while waiting for labour at the health centre. | HCP perspectives:  poor maternal healthcare seeking behaviour, specifically poor antenatal care attendance and giving birth at home.  physical barriers, respondents stated that long distances to the health centres, poor road network and non-availability of transport prevented many women from completing antenatal care visit schedules and giving birth at the clinic. |
| (Talbot et al., 2018) | Some were concerned that not only was the GP not the most appropriate person to initiate behaviour change discussions, but that the postnatal check was not the appropriate time. Two reasons given were a lack of time in the appointment to address behaviour change and a lack of readiness from women:  Some participants felt they lacked the resources and skills to discuss behaviour change effectively | They’re often totally worn out, they’re often not happy with the way they look ‘cause they’re often overweight, they’ve got stitching. Yeah I think it’s probably not the best moment to suggest major lifestyle changes.’ (GP18, Male [M]) p.e255:  ‘Obviously in the, in the relatively short time that the GP would have with that woman, I’m not quite sure I would necessarily be equipped enough anyhow to offer significant, sort of, you know, dietary advice, certainly, you know, I would mention it and ask about that and again signpost mum.’ (GP13, F) p.e256 |
| (Schmied et al., 2008) | **Midwives’ perceptions of quality postnatal care**  providing emotional and practical support for the woman and her family, enhancing a woman’s confidence in caring for her new baby and ability to make decisions, promoting women’s physical health and recovery particularly through rest, and monitoring the health of the baby.  Factors that facilitated quality care  There were several factors that participants suggested facilitated quality postnatal care. This included continuity of care and having a dedicated lactation support midwife.  **Factors that hindered quality care**  clerical tasks, such as the paper work associated with daily admissions and discharges Time. | providing emotional and practical support for the woman and her family, enhancing a woman’s confidence in caring for her new baby and ability to make decisions, promoting women’s physical health and recovery particularly through rest, and monitoring the health of the baby.  Many participants believed that the overemphasis on the daily postnatal physical check limited midwives’ ability to provide emotional and practical support and education.  They discussed the tensions that occurred between areas within the maternity unit and they also talked about contradictions in practices, such as trying to be flexible in the care provided and at the same time having following protocols and guidelines. |
| (Hyzam et al., 2020) | **Policy or system-related factors**   i) *Inadequate funding from government*   The public health facilities received inadequate funding from the government for the provision of basic and essential services.  **Provider-related factors**  **I*)****Inadequate human resource and medical equipment*  *Cost of services*  **Postnatal care was not necessary without complications.**  However, the discussions with the midwives demonstrated that PNC was also not provided at the health facilities for mothers after delivery. Further, midwives invited mothers whose pregnancies were somewhat marked with complications to attend the health facilities after delivery for a checkup. | System-related factors  **Provider-related factors**  **I*)****Inadequate human resource and medical equipment*  *Cost of services*  “In general, there is no physical examination for mothers after delivery at the reproductive health care centres. We [midwives] just provide blood sugar test for mothers who need sub-dermal contraceptive. Mothers come after delivery if they feel tired, her baby is sick, for vaccination, and family planning services” (Midwife 3; Lahj). P. 8  “I ask the mothers who had some health problems during the time of pregnancy to visit the health centres after childbirth to check their [mother] health status’. (Midwife 1; Abyan). P.9 |
| (Rayner et al., 2013) | Midwives commented they were useful at times, particularly for casual (non-permanent) staff, however the use of care plans often made the provision of individualised care more challenging; they felt preoccupied with completing the care plan rather than focusing on a woman’s individual needs:  While midwives reported educating women in the postnatal  period as a priority, they felt the volume of information, particularly the amount of predefined education listed on the care plan,  to be unrealistic and not individualised:  Although many postnatal education topics are usually covered during antenatal classes, midwives felt women are often unable to absorb this information, frequently commenting that women were more focused on pregnancy and birth  Staffing challenges. Midwives identified staffing issues as a significant barrier and a major constraint in providing quality postnatal care. They talked about levels of staffing often being inadequate and some of those interviewed felt that a shortage of midwives has resulted in increasing numbers of non-midwifery staff, such as mothercraft nurses, providing postnatal care. As one midwife commented:  Lack of continuity of care. Midwives highly valued the concept of continuity of care; however the structure of private obstetric maternity care, with obstetricians providing all antenatal care, makes achieving continuity prior to admission for labour and birth difficult. Unless women attend antenatal classes or a hospital tour, most will not meet any midwives until they arrive at the hospital in labour;  Further barriers to achieving continuity of care were reported to be due to a high proportion of midwives working part-time; the decreasing length of hospital stay; and some hospitals offering hotel care in which women change location and have part of their postnatal ‘admission’ in a hotel. Whilst acknowledging these difficulties, midwives attempted to establish continuity at least during the postnatal hospital stay.  Some midwives felt that hospital management viewed postnatal care as less of a priority compared with other aspects of maternity care and that this was reflected in inadequate staffing  \Others reported that they felt supported by management, and staff morale appeared better at hospitals in which midwives felt supported. | Care plans, CRAZY! They’ve driven me around the bend! It’s all just robotic! One same thing after another, the care plans are not individual, basically it’s not individualised. I probably should spend more time talking to the women (Clinical midwife, regional, 2009–3). P.624  I think they [the women] get loads, almost too much [information]yI think the women get confusedy. (Maternity unit manager, metropolitan, 2003–1). P.624  I actually think that postnatal information is not absorbed antenatally. That’s certainly my experience of twenty years. We are telling women exactly the same thing postnatally and it’s like they have never heard it before (Clinical midwife, metropolitan, 2003–1). P.624  Predominantly, we just have midwives but, we do have some mothercraft nurses just brought on staff, just to bolster the numbers slightly because there is a shortage of midwives and it’s been very hard to get new staff (Clinical midwife, metropolitan, 2019–1). P.624  ‘it’s quite possible that the women come in and they don’t even know where to go’ (Maternity unit manager, metropolitan, 2016–1).p.624  I think continuity of care is an enormous thing! I really like to try and follow women through the week, it makes a huge difference! (Clinical midwife, metropolitan, 2016–2). We try to create continuity of care for the women. It doesn’t always work because sometimes the skill mix and such means that you can’t (Maternity unit manager, metropolitan, 2003–1).p.625  . I suppose if you look at the fact that we struggle to get staff to give the care y you probably would say they, management, are not supportive (Clinical midwife, regional, 2009–3).p.626  Management are good! We actually [can] get women to come back after they’ve gone home, if we’re concerned about them (Maternity–2). P.626 |
| (Santos Junior et al., 2013) | **Limited professional exposure to PPD**  limited knowledge, awareness, and recognition of PPD by the health professionals.  Most participants reported no experiences in providing care for women with PPD. They viewed PPD as an uncommon problem; their focus of care was women’s physical wellbeing and ability to breastfeed.  **PPD is the domain of psychiatry**  Nurses and physicians described their reliance on others to identify PPD and care for women with PPD. Community health agents and women’s relatives brought cases of PPD to nurses’ and physicians’ attention.  **Challenges in dealing with PPD**  lack of comfort with approaching women who could potentially be suffering from PPD and lack of physical space for women to be treated. Discomfort with approaching women was layered on nurses’ and physicians’ lack of awareness of PPD. The healthcare providers’ difficulties, in part, arose from the absence of appropriate policies for assisting women with postpartum mood disorders. They had no clear protocols for engaging with women with PPD. | Here in our community we don’t have any cases of postpartum depression. I mean, either we don’t have any or we don’t know how to observe (n2). P.1252  Reliance on the others to identify PPD  lack of physical space for women to be treated.  lack of comfort with approaching women who could potentially be suffering from PPD  the absence of appropriate policies for assisting women with postpartum mood disorders |
| (Lugina et al., 2001) | **Reflecting**  Midwives reflected on the difficulties they faced in finding direction in their work because of a lack of guidelines. They also reflected on attitudinal problems and their readiness to accept deficiencies and act as change agents rather than waiting for directions all the time:  **Defining abilities**  ‘Defining abilities’ is the phrase used to describe the midwives’ desire and capability to know the areas in their professional practice that require improvement in terms of knowledge, attitudes and skills.  **Caring**  ‘Caring’ is the term that describes what the midwife does to assist the mother and the family in the postpartum period.  Caring included teaching mothers about self-care, focusing on prevention of infection. Attention to the psychological needs of the mother and need for rest were other aspects in care found to need priority in teaching. Another aspect of care was the need to assist couples to have good interpersonal and sexual relationships. Midwives felt they needed to assist couples to have realistic demands of each other:  Caring was also seen to include examining and touching and having good interpersonal relationship with clients. Postpartum women may have problems, not only due to lack of knowledge, but also because of midwives’ indifference to the individual needs of women | Lack of guidelines  the midwives’ desire and capability to know the areas in their professional practice that require improvement in terms of knowledge, attitudes and skills.  Caring included teaching mothers about self-care, focusing on prevention of infection.  Attention to the psychological needs of the mother. |
| (Mselle et al., 2017) | **Postpartum Education Is an Urgent Matter**  **Tensions Between Medical and Traditional Knowledge**  Many of the participants spoke about the tensions between medical and traditional knowledge particularly when mothers had to return to postnatal clinic after receiving remedies from family members that put them at risk. . . . and those who had Caesarean section and had anemia, we normally instruct them not to bathe using hot water when they go home. At home, they are looked after by their elderly relatives. They bathe them with hot water before the wound has properly healed, consequently, the wound gaps and bleeds. You may find that she was discharged with hemoglobin level of 8g/dl. By the time she comes back you find anemia is much serious. (NM)  One obstetrician wanted all mothers to have access to modern medical/health information.  they were not given time that might be needed to develop more trusting relationships or to discuss different ideas about postpartum issues such as breastfeeding or hygiene. The challenge is having adequate time to educate mothers because sometimes it is difficult for them to understand. For example, methods you use to make them understand . . . you may take even 2 hours to instruct them. You educate and if she goes out in a minute, she changes her mind because of the information she gets from other women. (OB)  Nurse-midwives and obstetricians were trying to lead the way as they struggled to negotiate and shift the binary relation of power and build trust with mothers and their families in the community. They said they needed more support.  **Including Family Members in Postpartum**  Education Participants in our study spoke about the importance of including family members during teaching sessions so that they could support the mother and baby at home with respect to many different aspects of care.  **Postpartum Educational Practices**  Nurse-midwives spoke about some of their educational practices such as assessing information recall or teaching in large groups. Both strategies were connected to their concern about imparting information in a short amount of time to many mothers to save lives and provide better quality of life for mothers and babies. For example, nurse-midwives described how they used “questioning” techniques to test whether the mothers understood what they had taught them. | tensions between medical and traditional knowledge particularly when mothers had to return to postnatal clinic after receiving remedies from family members.  No time to discuss different ideas about postpartum issues such as breastfeeding or hygiene  sometimes it is difficult for them to understand  difficult to make a trusted relationship with women.  F:  **Including Family Members in Postpartum**  Nurse-midwives spoke about some of their educational practices such as assessing information recall or teaching in large groups. |
| (Brodribb et al., 2013) | GPs thought that the majority of women who received shared antenatal care returned in the first seven to 10 days postpartum. However, they were less certain that other women who birthed in the public sector, returned.  I’d say about 60%… They’ve often been told that we’re busy; we haven’t got time [and] everything going fine. (Rural general practitioner (RGP 6).  They thought their responsibility extended to assessing how the family was functioning and providing anticipatory guidance and education when appropriate. For example, one GP said: You’ve got to be quite fluid....What is it that they’ve come to see you about? I’ve got to meet that need primarily. I’ve got to discharge my need which is keeping them safe and running through all the possible scenarios of something going terribly wrong… anything extra that I can plug in terms of education, that’s a bonus. (RGP7). P.6  **Content of a routine visit**  When discussing the content of a routine postpartum visit, the interviewed GPs mentioned undertaking a thorough examination of the infant, enquiring about infant feeding and the mother’s physical and mental wellbeing.  Appointment times ranged from 10 to 45–60 minutes with 30 minutes being common. The GPs acknowledged that the time allocated was often insufficient to manage problem with the mother or infant.  If you do pick up maternal issues, then that appointment can really blowout. (UGP4). P.6 | Less women return to see the GP who birthed in public sector  They thought their responsibility extended to assessing how the family was functioning and providing anticipatory guidance and education when appropriate.  time allocated was often insufficient to manage problem with the mother or infant. |
| (Rayner et al., 2008) | ***Midwives’ views of the aims of postnatal care***  midwives felt one of their primary roles during the postnatal stay was to educate women about breastfeeding and to provide them with information and support in this activity.  Aiding women’s physical recovery from pregnancy and birth Related to their educative role, midwives spoke of promoting maternal health and the importance of educating and informing women about normal physiological changes to their bodies after birth and breastfeeding. Pregnancy and birth was viewed as a normal life stage for women, but education included enabling women to identify any health problems, such as mastitis or a wound infection that may arise after discharge.  *Organisational barriers to postnatal care*  the length of the postnatal stay for most women; the busyness and chaotic nature of maternity units; the inadequacy of staffing especially at night; and the effect large numbers of visitors had on postnatal education and care:  So there’s a heck of a lot of issues, and unfortunately, I think that the postnatal comes down as being a poor cousin to the others. (KI 1012-1, Regional) (p.316) | Breastfeeding was the main concern to educate women.  Pregnancy and birth was viewed as a normal life stage for women, but education included enabling women to identify any health problems, such as mastitis or a wound infection that may arise after discharge.  ***Organisational barriers to postnatal care***  the length of the postnatal stay for most women; the busyness and chaotic nature of maternity units; the inadequacy of staffing especially at night; and the effect large numbers of visitors had on postnatal education and care |
| (Olsson et al., 2011) | **Balancing between personal perceptions of the woman’s needs and health system restrictions** |  |
| (Forster et al., 2006) | **The impact of staffing on the quality of postnatal care**  The key informant interviews gave some insight into the potential negative impacts arising in relation to staffing issues and the provision of quality postnatal care. The broad themes that arose were adequacy of staff/patient ratios, staff stress and issues around continuity of care versus core staffing.  *Core staffing versus continuity models*  very positive about models that provided for increased continuity of care by a system of  staff rotation, and negative toward core staff, or staff who were reluctant to work in all areas. Two key informants said that they had tried to introduce rotations or team care in their unit to enhance continuity, but it just did not work in their particular situation. The issue of postnatal staff often being moved to other areas that are short of staff (sometimes just after a midwife has met the women and told then she will be caring for them) also impacts on continuity in postnatal wards:  *Staff-patient ratios*  *Staff distress related to adequacy of staffing*  *Being in a rural location*  Many rural respondents commented that being located in a rural area was a significant contributing factor to staff recruitment difficulties:  *Midwifery skill maintenance*  Rural midwives in particular may have difficulty in maintaining their skill levels due to low birth numbers, as well as the 'low risk' status of many of the women for whom they provide care. There may often be limited opportunities to maintain the skills required to care for emergencies or women of higher 'risk'. | adequacy of staff/patient ratios, staff stress and issues around continuity of care versus core staffing.  continuity of care by a system of  staff rotation, and negative toward core staff, or staff who were reluctant to work in all areas.  Shortage of staff  *Staff-patient ratios*  *Staff distress related to adequacy of staffing*  *Being in a rural location*  As with many rural hospitals it's difficult to attract new midwives unless they already have ties to this area. Retention does  not appear to be an issue here (survey-1041, rural)  Recruitment usually an ongoing issue due to the hospital being  in a rural area (survey-1028, rural)  The issue of recruiting and retaining midwives in rural  areas was also reported to be related to employment  opportunities for the midwives' partners:  Geographical isolation [is an issue, with] lack of employment  opportunities for spouses (survey-1074, regional)  Retention often depends on their partner's job too (survey-  1047, regional) p.9  difficulty in maintaining their skill levels due to low birth numbers, as well as the 'low risk' status of many of the women for whom they provide care.  There may often be limited opportunities to maintain the skills required to care for emergencies or women of higher 'risk'. |
| (Alnuaimi et al., 2020) | **Silence Culture and tradition/embarrassing topic**  embarrassment among Jordanian women was the main reason behind the lack of educational discussion regarding sexuality.  women did not ask about sexuality because they were embarrassed to initiate a conversation with HCPs.  **Poor communication**  Despite that care professionals’ awareness of women embarrassment, they did not offer a discussion to encourage them to talk.    the work overload as another reason hindering them from discussing this topic. A nurse said: “It is because of the workload … we don’t have time to speak with them.” A midwife also reported: “we don’t ask women because we don’t have enough time to finish our tasks.”  Culture, norms, and beliefs of Jordanian women were considered as another barrier that inhibits providing appropriate health care education. HCPs avoid discussing such things because it is not acceptable and might be seen as crossing borders. A midwife said: “Sometimes our culture and norms inhibit the communication between our clients and us as they considered opening this issue is a shame and forbidden … so I think if we start the chat, women will accept the discussion and be encouraged to ask questions”. (p.642)  **Unclear responsibility and lack of knowledge**  Many participants believe that it was not their responsibility to discuss such issue, a nurse reported: **“I never discuss this topic with my clients … .it is not my job**”. Another nurse highlighted the poor documentation regarding this topic in their job description and stated: “**I think discussing this issue is not included in my job description or responsibilities**”.  However, others see education about sexuality is not one of the postpartum topics that should be discussed with women, a midwife stated: **“I’m not sure if this subject is one that I should teach women about … .”.** A nurse criticized the nursing courses that lacked information about this topic either during studying time or during working at the health centre, which yielding insufficient knowledge. “**Nobody told me to discuss this topic with women I do not know too much about this topic**”.p.6  **suggestions for sexual education approaches**  Our research participants suggested giving written materials at antenatal clinics to all women then they can pass this information to their husbands.  “I think offering written materials about sexuality would be a good idea, then they can discuss the issue with their husbands at home”. (P.644)  suggested establishing a counselling clinic to provide advice and consultations to all clients. “Consultation for sexuality in a private office would be a good idea to help all clients especially for people who feel embarrassed to share such subject”.  The last suggestion was using the social media that is widely used:  “the best thing is to contact people through the social media … most people nowadays like using smartphones and enjoy reading online websites … I know many websites with consultants, men or women can ask any question they want”. (P.645) | “really … . why I never open this topic with women?” “It never comes to my mind to ask about such thing (sexuality issue)” (p.642) |
| (Pallangyo et al., 2017) | **We** **could do better if we were organized, knowledgeable, supervised and had resources.**  The areas for improvement envisioned by the HCP in the FGD included: **organization of space, time, resources, communication and referral system; improvement of their own PPC knowledge; and supervision and feedback.**  It was also advocated that PPC should be practiced every day and at all times. That would help reduce the waiting times for mothers and fathers and motivate them to attend PPC services. Uncommitted colleagues who spent most of their time in activities not related to clients were described as problematic.  Understaffing was also brought forward as a factor that hinders quality care.  Appropriate training could increase the providers’ confidence and performance, improve the care, diminish misdiagnosing and contribute to saving lives. Sharing of knowledge between professionals and working more closely together could also add to the quality of care.  Updates on recent research and new evidence would add to their confidence and their use of evidence-based practice.  They argued that if they decide to bring about change, there is a possibility for betterment despite the existing obstacles. |  |
| (Pope et al., 2023) | **Supporting women in healthcare settings**  Building relationships to provide support and advocacy  Support women and listen to them as a factor to attract women  **Women can get “lost in the system”**  *Concerns about training, skills, and scope:* . Lack of time, and other professional demands, were identified as barriers to attending these optional trainings.  HCPs also reported limited confidence in selecting or facilitating appropriate mental health interventions.  *Looking beyond medicalised approaches*  HCPs were wary about pathologising or taking a medicalised approach to understanding and treating women’s mental health concerns.  Some HCPs perceived that the emphasis on clinical disorders in healthcare training came at the expense of educating providers about more common issues in mental health and well-being. In addition, many HCPs regarded perinatal-specific stress as common and “natural”  **Structural barriers to care provision**  high patient volume and care ratios, the lack of privacy on wards and in appointment spaces, and a culture which pressured them to be “running around physically working” rather than spending time with women.  Additionally, some midwives reported that the quality of medical decision-making was influenced by dysfunctional power dynamics on care teams, with potentially harmful psychological consequences for women.  *Insufficient organisational investment and resources*  inadequacy of existing referral pathways, particularly for women without private health insurance. Long waitlists for public mental healthcare, and the lack of public services tailored to the physical, scheduling, and childcare needs of new mothers, left many women without timely access to support.  *Social barriers to support*  social inequities in access to perinatal mental health supports.  disparities between the mental health services available in different regions and facilities.  social factors, such as language or socioeconomic status, could impact relationships between women and medical staff, with consequences for the support women received. For example, many HCPs found it challenging to detect mental health issues for women with limited English language proficiency due to lack of interpreters.  difficulties developing and maintaining relationships with women from lower socioeconomic backgrounds due to point-of-service fees in primary care, and challenges in facilitating mental health disclosures from women from lower socioeconomic backgrounds due to these women’s concerns about differential targeting in child protective investigations. |  |
| (Verma & Mukherjee, 2020) | **Manpower Linked**  Shortage of Nurses  Shortage of specialist doctor,  **Facility Linked:**  Infrastructure  Lack of diagnostic facilities creates difficulty in the decision-making process in obstetric emergency cases.  **Security and safety threats:**  No provision of financial incentive for higher education: Nurses did not get any financial incentive after acquiring higher education.  Ineffective patient-provider communication  **Limited internet connectivity:**  Limited network connectivity was a major barrier perceived by the participants. |  |
| (Martin et al., 2014) | **(a) lack of women’s knowledge about postpartum health and lack of preparation**  **(b) lack of continuity of care and absence of maternal care during the early postpartum period.**  Clinicians also acknowledged that care continuity was important in patient-provider communication during the postpartum visit.  Clinicians mentioned that maintaining the same provider in prenatal care through the postpartum period allowed providers to tailor postpartum care to the needs of the patients, rather than review a general checklist of items Clinicians mentioned that maintaining the same provider in prenatal care through the postpartum period allowed providers to tailor postpartum care to the needs of the patients, rather than review a general checklist of items  **(c) disconnect between providers and postpartum**  **Mothers ********  **d) bridging the gap: suggestions for**  **Improvement** | When you see them and you’ve been seeing them, it’s  a continuation of a discussion you’ve been having for  nine months. [MD provider]  …if the patient knows you and feels comfortable with you, they’re just gonna talk to you and say ‘Hey this is bugging me’ or whatever. But if the patient doesn’t know you, she’s just kinda… sitting there, waiting for you to ask… [MD provider] |
| (Bains et al., 2023) | **Theme 1: Lack of awareness and understanding of perinatal mental illness and service structure**  **in both healthcare professionals and patients**  Cultural and spiritual attributions:  **Theme 2: Patients’ relationships with family, friends and healthcare professionals can both hinder and facilitate access to services.**  **Theme 3: Healthcare professionals promote raising awareness, flexibility, developing shared understandings**  **and questioning assumptions to improve the accessibility and acceptability of services**  **Work towards shared meanings between HCPs and patients:**  **“There’s a different type of understanding of mental health**  **and what mental health means to some cultural groups”** | I’ve seen quite a lot of mums from that background that live in denial. They don’t seek help. There are certain cultural beliefs. Most mums in that position, from where they come from, and like where I come from, they don’t like to accept that they have that depression. They don’t believe in it.—HCP017  (Health Visitor) p.4  “[Patients] don’t like to accept that they have depression, they don’t believe  in it”.  Family can prevent people from accessing help. I think that is something that’s come up in sessions, an idea that it’s helpful to keep things within family and that you sort things out within your family and that’s it.—HCP002  (Psychologist) …two out of the eight ladies that I spoke to had difficulties in accessing mental health, purely because  their family didn’t agree with them accessing any mental health services. It’s OK if it was pregnancy, it was OK if it was medical, but mental health wise,  they were kind of being discouraged away from doing that.—HCP008 (Midwife) p. 6  maybe we do need more culturally sensitive information or awareness to be aimed specifically for communities. And it’s whether those communities.  would be accepting of a big public sort of awareness campaign, or actually it’s better aimed in more local, than national… I don’t know whether… targeting this in a more local way is more appropriate, acceptable.—HCP008 (Midwife) p.9 |
| (Poon et al., 2021) | **Personal attributes towards postpartum care**  **Gender**  The PCPs identified the gender of the physician to be a significant factor influencing the consultation agenda  with a postpartum mother. As postpartum care involved examination of the more private areas, such as the genital and the breasts, male PCPs reflected that they were less likely to be consulted by postpartum women. The latter might not bring up the related issues even if they were attended by male PCPs.  **Challenges relating to the healthcare system and policies Limited consultation time and ineffectual counter‑measure**  One common barrier pertaining to the comprehensive postpartum reviews highlighted by the PCPs was the  inadequate consultation time allocated for a postpartum visit in primary care setting. Most felt that a more realistic time per consult should last from 15 to 20 min, compared to the current 5 to 10  min in real-time clinical practice. The limited time for consult often reduced the comprehensiveness and scope of postpartum issues covered within the postpartum visit. Consequently, the  PCPs tended to avoid time-consuming tasks such as mental health assessment or discussing contraception options with the mothers. To overcome the short consult, some PCPs advised mothers to return for a separate visit  to tie up loose ends. However, the PCPS recognised that default rate would be high, resulting in incomplete resolution of issues faced by postpartum mothers. | “Some of them, they prefer a female doctor instead of a male doctor, especially if it involves a lot of checking (of) the breast and also the private part(s)” (PC6, Male Medical Officer in Polyclinic)“My credibility is definitely less than hers. She (female colleague) breastfed four children of her  own. I can’t breastfeed any of my children. I have no credential.” (PC25, Male GP) p.5  “It’s actually time that is given to us. If these patients turn up in general pool (referring to walk-in consultation), …, within 6-min consult, it’s very, very  challenging to address (the) so many aspects of the maternal health” (PC2, Female Family Physician in  Polyclinic) p.7 |
| (Ruderman et al., 2021) | **Environmental factors**  Environmental factors influencing the ability of individuals to access care were defined as factors related  to the broader environment outside of the health care setting. Subthemes of environmental barriers included  housing instability, limited childcare, and transportation challenges.  Only one environmental facilitator of postpartum care was identified (Table 4). Specifically, many providers recommended coordinating postpartum and newborn care as an effective way to overcome some of  these barriers.  **Societal factors**  Societal factors related to a patient’s ability to access optimal health care at an affordable **cost** as well as  the influence of groups in their community on their antepartum and postpartum experience. Subthemes included insurance limitations and lack of access to a primary care provider.  **Institutional factors**  Institutional issues addressed barriers or facilitators to effective patient care within the hospital system and  clinic environment.  Providers described appointment  scheduling difficulties, short appointment length, inability to place referrals within the system, lack of continuity, nonclinical task burden, and training gaps as notable barriers.  **Interpersonal factors**  Interpersonal dynamics were described as those for which health care use and outcomes were directly influenced by people. Interpersonal barriers centered on patient relationships and included lack of trust with providers, language barriers, concern for patient autonomy, limited.  family support, and a focus on the pregnancy rather than the patient.  Multiple interventions to improve care at the interpersonal level were identified (Table 4). These included.  counseling on the importance of follow-up, continuity with providers, clearer guidelines of when patients  should return, and telemedicine to facilitate more frequent follow-up.  *Telemedicine to facilitate more frequent follo*w-up as a facilitator:  **Interpersonal factors**  Interpersonal dynamics were escribed as those for which health care use and outcomes were directly influenced by  people. Interpersonal barriers entered on patient relationships and included lack of trust with providers, language barriers, concern for patient autonomy, limited family support, and a focus on the pregnancy rather than the patient. Mistrust in unfamiliar providers along with mistrust of the medical system at large, as well as language barriers were major concerns. The need to balance providers’ facilitation of care with patient autonomy was a theme central to this discussion. Providers reported a tension in which the concern was not that they could not do more, but that they should not do more for patients in terms of care coordination and follow-up. One provider described the tension as:  **Individual factors**  Individual concerns were defined as issues related to patient and providers directly without influence of other people or institutions. Individual-level barriers that providers ascribed to patients included asymptomatic disease processes, low health literacy, and postpartum mental health issues (Table 3).  Many providers expressed frustration at patients who did not follow-up due to ‘‘feeling fine,’’ one stating that:  Regarding low health literacy, providers often cited a lack of patient understanding of health conditions. For example, one physician described a patient who did not understand her preeclampsia diagnosis and endorsed a lack of ‘‘understanding what that even means, what the name  of the disease is and what the symptoms associated are.’’ Postpartum mental health issues, most notably postpartum depression, were also perceived as a factor  that limited patient engagement in care (Table 3).  Facilitators for improving postpartum care included increased educational resources and making clear the  tangible gains from attending appointments (Table 4).  Examples of informational resources included handouts, discharge instructions, websites, or other information from health care team members. Tangible gains included contraception, blood pressure checks, or participation in a peripartum mood disorder program offered  at this clinical site. One provider aptly described their belief about why patients return to postpartum care: | ‘‘Oftentimes when I have patients who don’t show. the most common reasons that I hear are I didn’t have a way to get to the clinic or I didn’t have anyone  to watch my kids.’’ P.256  ‘‘I think something that could be done to help facilitate that is like if we could coordinate even with like the baby’s care so that if the mom was gonna come to the pediatrician’s visit, like we could do the blood pressure check that day.’’ P.256  ‘‘Patients navigating their insurance for the first time whether they are uninsured and now because of the  pregnancy have become eligible to receive Medicaid and navigating that whole process can be very  challenging for patients.’’  p.257  ‘‘I think our access for a social worker is really great and she is very very on top of it about being in communication.’’p.258  ‘‘I think people engage when they have an issue but other than that they certainly are less likely to come to their postpartum visit.’’ P.258  ‘Patients who have been [seen] by the same provider and they’ve been lucky enough to get that continuity and they know that they’re going to go back and see that same provider again in the future, that’s an increased likelihood that they’re going to return.’ P.258  ‘‘I think it’s something that has been really nice about this period of time is like actually being able to call a patient and feeling empowered to do that.’’p.258  ‘‘I also personally struggle with the push and pull between giving patients their autonomy as they’re adults who are taking care of kids and have their own schedules to deal with, and then us making appointments for them. I don’t know whether that’s actually helping or not.’’ P.259  ‘‘A lot of times patients just come back because they want an IUD or they need like a contraception plan if they’re engaged in care but otherwise [without those tangible benefits] I think they feel like they’re healthy enough and now their focus is the baby.’’ P.260  ‘‘I definitely had a patient come in after six weeks for a complaint who was like. I was feeling healthy at six weeks, so I didn’t come in.’’  ‘‘A lot of times patients just come back because they want an  IUD or they need like a contraception plan if they’re engaged in care but otherwise [without those tangible benefits] I think they feel like they’re healthy enough and now their  focus is the baby.’’ P.260 |
| (Weldearegay et al., 2020) | work related burnout, gap between providers’ skills and knowledge, being fear of litigation, poor motivation schemes and issues related  to their retention, shortage of SBAs mainly midwives, lack of authority to make decisions, unable to translate training into practice and unavailability of adequate medications and necessary equipment were important reasons for poor quality of care during childbirth and immediate postpartum care. These issues are reflected in a series of quotations highlighted herein: | While poor motivation and satisfaction pressures due to low salaries  and allowance were found to greatly affect the care SBAs delivered,  they were not the only principal barriers. Fear of law suit, shortage  of human resource, unhygienic infrastructure and inadequate  availability of medicine and supplies impact up on quality practice  (KII participant, unit head of maternity ward).  . . . heavy work load leads providers’ burn out resulting from insufficient  and can lead to poor performance, inappropriate behavior and attitude.  In addition, limited proper capacity building devices and incompetent  SBAs remain serious barriers to provide quality of maternity care  services in Tigray (KII participant, Woreda MCH expert).  Despite much training conducted so far, whatever their role is significant in improving knowledge and skill of providers, but should have to be supplemented through coaching and mentoring which in turn increased their level of confidence in delivering services and enabled them to increase adherence to good practice and  standards.” (FGD participant, diploma midwife) Almost all SBAs have a fear of litigation in provision of maternal and  newborn care services. Thus, have a legal protection of SBAs in terms  of medical indemnity insurance is important to apply their highest  level potential in reducing unnecessary referral and averting maternal and  newborn death. (KII participant, hospital medical director) p.16 |
| (Akongo et al., 2024) | Key informants indicated that most mothers lack of transport to the health facility is also a barrier. Being financially restrained, mothers fail to access the health facility and yet they need the services.  Most of the health workers at the postnatal and young child clinic are not well informed about postpartum depression and do not think its common except for the few who recognize the symptoms of PPD.  The health workers have limited time to spend with each mother considering the huge volume of patients they have to attend to, so they normally focus on physical health only.  Participants noted that most mothers don´t present to the health facility because they opt to seek care from the traditional healers and herbalists within their communities. Also, because there are no formal linkages between mothers who deliver from home and health facilities, most of them tend to visit health facilities only when psychological distress is significant. In addition, given the symptoms of postpartum depression, some people mistake it for something else and they choose inappropriate care.  Facilitators for accessing postpartum care    However, participants indicated that psychosocial support at the postnatal clinic has greatly helped them. Counselling is normally done at the postnatal clinics to the women who seek for help. Women who receive routine psychological support have more reasons to access care because they have backup support. The FGD pointed out that women receive psychosocial support from their partners, families, friends and healthcare providers and this support gives them relief and encourages them to seek for more help when experiencing psychological distress. Most women get relieved when counseled. And the health care providers offer encouragement to the mothers facing psychological distress.    Health workers give health education in the morning at the postnatal clinic and mothers who show some psychological distress are linked to care for further management. Mothers who present with symptoms of postpartum depression and are noticed by the healthcare workers are linked to the mental unit for screening and treatment where need be. The health care providers in postnatal and young child clinics talk to the mothers´ experiencing psychological distress and link them to the mental health unit for proper care. This facilitates the mothers to seek more care. | *“ … we take it that those mothers who have not come back for review have issues that stopped them and mostly it is lack of transport.”* (KI 003)  *“Postpartum depression, is it common in postnatal hmmmm maybe they take long to regain and you can check the hemoglobin levels (HB) and take off blood sample”* (KI postnatal clinic)  *“While we would love to handle every patient well, this is not feasible because of time constraints. Here we see very many mothers and when you think of giving each one enough time to talk, the numbers pile up and you may not be able to attend to all the patients and accomplish all the day´s tasks. Haaa women are many in this facility”* . (KI from postnatal clinic)  *… they first start with the natives such as herbalists before they come here* (KI from young child clinic).    *“Where these mothers deliver from affects their health-seeking behaviour. Because those who deliver from home and experience mental health distress symptoms, may believe that these symptoms are a nonbiomedical illness, they seek care from traditional healers or any other place of their choice”*   “They counsel you and what was worrying you seems to be lightened. Even when you had something in mind like taking the child to the father and leaving it there, you may change your mind and take care of it” (FGD 002).  (KI from me“  We encourage the mothers to confide in and seek support from their friends and relatives and if they were working, we also talk to their employers” (KI from mental unit)  ntal unit)  “When we talk to the mother and her attendant and then link her to the mental ward, there I think she is managed well, given drugs then sent back home” . (KI from postnatal unit) |

**References:**

Akongo, D., Gwokyalya, V., Tusubira, A., Waiswa, P., Wamala, K., Tuhebwe, D., Kisakye, A. N., & Namutundu, J. (2024). Barriers and facilitators to seeking postpartum depression care services among postpartum mothers in Jinja Hospital Uganda. *Journal of Interventional Epidemiology and Public Health*, *7*(20).

Alnuaimi, K., Almalik, M., Mrayan, L., Mohammad, K., Ali, R., & Alshraifeen, A. (2020). "Resumption of Sexuality and Health Education in Postpartum Period: From Jordanian Health Care Professionals' Perspectives". *Journal of Sex & Marital Therapy*, *46*(7), 639-648. <https://doi.org/10.1080/0092623x.2020.1769242>

Bains, K., Bicknell, S., Jovanović, N., Conneely, M., McCabe, R., Copello, A., Fletcher-Rogers, J., Priebe, S., & Janković, J. (2023). Healthcare professionals’ views on the accessibility and acceptability of perinatal mental health services for South Asian and Black women: a qualitative study. *BMC Medicine*, *21*(1), 370. <https://doi.org/10.1186/s12916-023-02978-5>

Brodribb, W., Zadoroznyj, M., & Dane, A. (2013). The views of mothers and GPs about postpartum care in Australian general practice. *BMC Fam Pract*, *14*, 139. <https://doi.org/10.1186/1471-2296-14-139>

Forster, D. A., McLachlan, H. L., Yelland, J., Rayner, J., Lumley, J., & Davey, M. A. (2006). Staffing in postnatal units: is it adequate for the provision of quality care? Staff perspectives from a state-wide review of postnatal care in Victoria, Australia. *BMC Health Services Research*, *6*, Article 83. <https://doi.org/10.1186/1472-6963-6-83>

Hyzam, D., Zou, M. Y., Boah, M., Saeed, A., Li, C. R., Pan, S. X., Zhai, J. H., & Wu, L. J. (2020). Health information and health-seeking behaviour in Yemen: perspectives of health leaders, midwives and mothers in two rural areas of Yemen. *Bmc Pregnancy and Childbirth*, *20*(1), Article 404. <https://doi.org/10.1186/s12884-020-03101-9>

Lugina, H. I., Lindmark, G., Johansson, E., & Christensson, K. (2001). Tanzanian midwives' views on becoming a good resource and support person for postpartum women [Article]. *Midwifery*, *17*(4), 267-278. <https://doi.org/doi:10.1054/midw.2001.0285>

Lundeen, T., Musange, S., Azman, H., Nzeyimana, D., Murindahabi, N., Butrick, E., & Walker, D. (2019). Nurses' and midwives' experiences of providing group antenatal and postnatal care at 18 health centers in Rwanda: A mixed methods study [Research Support, Non-U.S. Gov't]. *PLoS ONE [Electronic Resource]*, *14*(7), e0219471. <https://ezproxy.utas.edu.au/login?url=http://ovidsp.ovid.com/ovidweb.cgi?T=JS&CSC=Y&NEWS=N&PAGE=fulltext&D=med16&AN=31295335>

<http://rk9dr6cc2p.search.serialssolutions.com/?url_ver=Z39.88-2004&rft_val_fmt=info:ofi/fmt:kev:mtx:journal&rfr_id=info:sid/Ovid:med16&rft.genre=article&rft_id=info:doi/10.1371%2Fjournal.pone.0219471&rft_id=info:pmid/31295335&rft.issn=1932-6203&rft.volume=14&rft.issue=7&rft.spage=e0219471&rft.pages=e0219471&rft.date=2019&rft.jtitle=PLoS+ONE+%5BElectronic+Resource%5D&rft.atitle=Nurses%27+and+midwives%27+experiences+of+providing+group+antenatal+and+postnatal+care+at+18+health+centers+in+Rwanda%3A+A+mixed+methods+study.&rft.aulast=Lundeen>

Martin, A., Horowitz, C., Balbierz, A., & Howell, E. A. (2014). Views of Women and Clinicians on Postpartum Preparation and Recovery. *Matern Child Health J*, *18*(3), 707-713. <https://doi.org/10.1007/s10995-013-1297-7>

Mselle, L. T., Aston, M., Kohi, T. W., Mbekenga, C., Macdonald, D., White, M., Price, S., Tomblin Murphy, G., O’Hearn, S., & Jefferies, K. (2017). The Challenges of Providing Postpartum Education in Dar es Salaam, Tanzania: Narratives of Nurse-Midwives and Obstetricians. *Qualitative Health Research*, *27*(12), 1792-1803. <https://doi.org/10.1177/1049732317717695>

Olsson, A., Robertson, E., Falk, K., & Nissen, E. (2011). Assessing women's sexual life after childbirth: the role of the postnatal check [Research Support, Non-U.S. Gov't]. *Midwifery*, *27*(2), 195-202. <https://ezproxy.utas.edu.au/login?url=http://ovidsp.ovid.com/ovidweb.cgi?T=JS&CSC=Y&NEWS=N&PAGE=fulltext&D=med8&AN=19570597>

<http://rk9dr6cc2p.search.serialssolutions.com/?url_ver=Z39.88-2004&rft_val_fmt=info:ofi/fmt:kev:mtx:journal&rfr_id=info:sid/Ovid:med8&rft.genre=article&rft_id=info:doi/10.1016%2Fj.midw.2009.04.003&rft_id=info:pmid/19570597&rft.issn=0266-6138&rft.volume=27&rft.issue=2&rft.spage=195&rft.pages=195-202&rft.date=2011&rft.jtitle=Midwifery&rft.atitle=Assessing+women%27s+sexual+life+after+childbirth%3A+the+role+of+the+postnatal+check.&rft.aulast=Olsson>

Pallangyo, E. N., Mbekenga, C., Kallestal, C., Rubertsson, C., & Olsson, P. (2017). "If really we are committed things can change, starting from us": Healthcare providers' perceptions of postpartum care and its potential for improvement in low-income suburbs in Dar es Salaam, Tanzania. *Sexual & reproductive healthcare : official journal of the Swedish Association of Midwives*, *11*, 7-12. <https://ezproxy.utas.edu.au/login?url=http://ovidsp.ovid.com/ovidweb.cgi?T=JS&CSC=Y&NEWS=N&PAGE=fulltext&D=med14&AN=28159132>

<http://rk9dr6cc2p.search.serialssolutions.com/?url_ver=Z39.88-2004&rft_val_fmt=info:ofi/fmt:kev:mtx:journal&rfr_id=info:sid/Ovid:med14&rft.genre=article&rft_id=info:doi/10.1016%2Fj.srhc.2016.09.001&rft_id=info:pmid/28159132&rft.issn=1877-5756&rft.volume=11&rft.issue=&rft.spage=7&rft.pages=7-12&rft.date=2017&rft.jtitle=Sexual+%26+reproductive+healthcare+%3A+official+journal+of+the+Swedish+Association+of+Midwives&rft.atitle=%22If+really+we+are+committed+things+can+change%2C+starting+from+us%22%3A+Healthcare+providers%27+perceptions+of+postpartum+care+and+its+potential+for+improvement+in+low-income+suburbs+in+Dar+es+Salaam%2C+Tanzania.&rft.aulast=Pallangyo>

Poon, Z., Lee, E. C. W., Ang, L. P., & Tan, N. C. (2021). Experiences of primary care physicians managing postpartum care: a qualitative research study. *BMC Family Practice*, *22*, 1-12.

Pope, J., Redsell, S., Houghton, C., & Matvienko-Sikar, K. (2023). Healthcare professionals’ experiences and perceptions of providing support for mental health during the period from pregnancy to two years postpartum. *Midwifery*, *118*, 103581.

Rayner, J. A., Forster, D., McLachlan, H., Yelland, J., & Davey, M. A. (2008). A state-wide review of hospital postnatal care in Victoria, Australia: The views and experiences of midwives. *Midwifery*, *24*(3), 310-320. <https://doi.org/10.1016/j.midw.2006.10.008>

Rayner, J. A., McLachlan, H. L., Peters, L., & Forster, D. A. (2013). Care providers' views and experiences of postnatal care in private hospitals in Victoria, Australia [Research Support, Non-U.S. Gov't]. *Midwifery*, *29*(6), 622-627. <https://ezproxy.utas.edu.au/login?url=http://ovidsp.ovid.com/ovidweb.cgi?T=JS&CSC=Y&NEWS=N&PAGE=fulltext&D=med10&AN=23123157>

<http://rk9dr6cc2p.search.serialssolutions.com/?url_ver=Z39.88-2004&rft_val_fmt=info:ofi/fmt:kev:mtx:journal&rfr_id=info:sid/Ovid:med10&rft.genre=article&rft_id=info:doi/10.1016%2Fj.midw.2012.05.006&rft_id=info:pmid/23123157&rft.issn=0266-6138&rft.volume=29&rft.issue=6&rft.spage=622&rft.pages=622-7&rft.date=2013&rft.jtitle=Midwifery&rft.atitle=Care+providers%27+views+and+experiences+of+postnatal+care+in+private+hospitals+in+Victoria%2C+Australia.&rft.aulast=Rayner>

Ruderman, R. S., Dahl, E. C., Williams, B. R., Feinglass, J. M., Grobman, W. A., Kominiarek, M. A., & Yee, L. M. (2021). Provider perspectives on barriers and facilitators to postpartum care for low-income individuals. *Women's Health Reports*.

Santos Junior, H. P. O., Rosa Gualda, D. M., de Fátima Araújo Silveira, M., & Hall, W. A. (2013). Postpartum depression: the (in) experience of B razilian primary healthcare professionals. *Journal of Advanced Nursing*, *69*(6), 1248-1258.

Schmied, V., Cooke, M., Gutwein, R., Steinlein, E., & Homer, C. (2008). Time to listen: strategies to improve hospital-based postnatal care. *Women and Birth*, *21*(3), 99-105.

Sialubanje, C., Massar, K., Hamer, D. H., & Ruiter, R. A. (2014). Understanding the psychosocial and environmental factors and barriers affecting utilization of maternal healthcare services in Kalomo, Zambia: a qualitative study [Research Support, Non-U.S. Gov't]. *Health Education Research*, *29*(3), 521-532. <https://doi.org/https://dx.doi.org/10.1093/her/cyu011>

Talbot, H., Strong, E., Peters, S., & Smith, D. M. (2018). Behaviour change opportunities at mother and baby checks in primary care: a qualitative investigation of the experiences of GPs. *British Journal of General Practice*, *68*(669), e252-e259. <https://ezproxy.utas.edu.au/login?url=http://ovidsp.ovid.com/ovidweb.cgi?T=JS&CSC=Y&NEWS=N&PAGE=fulltext&D=med15&AN=29530920>

<http://rk9dr6cc2p.search.serialssolutions.com/?url_ver=Z39.88-2004&rft_val_fmt=info:ofi/fmt:kev:mtx:journal&rfr_id=info:sid/Ovid:med15&rft.genre=article&rft_id=info:doi/10.3399%2Fbjgp18X695477&rft_id=info:pmid/29530920&rft.issn=0960-1643&rft.volume=68&rft.issue=669&rft.spage=e252&rft.pages=e252-e259&rft.date=2018&rft.jtitle=British+Journal+of+General+Practice&rft.atitle=Behaviour+change+opportunities+at+mother+and+baby+checks+in+primary+care%3A+a+qualitative+investigation+of+the+experiences+of+GPs.&rft.aulast=Talbot>

Verma, M., & Mukherjee, K. (2020). Perceptions of nurses regarding barriers to implement maternal and newborn care at rural community health centers of Madhya Pradesh, India: A phenomenology study. *Indian Journal of Public Health Research and Development*, *11*(2), 671-676. <https://ezproxy.utas.edu.au/login?url=http://ovidsp.ovid.com/ovidweb.cgi?T=JS&CSC=Y&NEWS=N&PAGE=fulltext&D=emexb&AN=2004452572>

<http://rk9dr6cc2p.search.serialssolutions.com/?url_ver=Z39.88-2004&rft_val_fmt=info:ofi/fmt:kev:mtx:journal&rfr_id=info:sid/Ovid:emexb&rft.genre=article&rft_id=info:doi/10.37506%2Fv11%2Fi2%2F2020%2Fijphrd%2F194885&rft_id=info:pmid/&rft.issn=0976-0245&rft.volume=11&rft.issue=2&rft.spage=671&rft.pages=671-676&rft.date=2020&rft.jtitle=Indian+Journal+of+Public+Health+Research+and+Development&rft.atitle=Perceptions+of+nurses+regarding+barriers+to+implement+maternal+and+newborn+care+at+rural+community+health+centers+of+Madhya+Pradesh%2C+India%3A+A+phenomenology+study&rft.aulast=Verma>

Weldearegay, H. G., Kahsay, A. B., Medhanyie, A. A., Godefay, H., & Petrucka, P. (2020). Quality of and barriers to routine childbirth care signal functions in primary level facilities of Tigray, Northern Ethiopia: Mixed method study. *PLoS One*, *15*(6), e0234318.
