# Supplementary material for: Barriers and Facilitators to Providing Women Postpartum Care by Health Care Providers in the First 12 Months After Childbirth: A Systematic Qualitative Meta‐Aggregation Review
Source: J Midwifery Womens Health. 2026 Jun 16;71(5):820–40. doi: 10.1111/jmwh.70142 (PMC13655192; doi:10.1111/jmwh.70142)
Supplement: Supplementary file 2 [file JMWH-71-820-s002.docx]

| **Concept** | **Search Terms** | **Boolean/Truncation Used** |
| --- | --- | --- |
| **Type of Care** | Midwifery care OR Maternal care OR Care | OR |
| **Postpartum Period** | Postpartum OR post-partum OR Perinatal OR Postnatal OR Parturition OR After childbirth OR After birth OR Following birth OR Puerperium | OR |
| **Healthcare Providers** | General Practitioners OR Midwife OR Maternal and Child Health Nurses OR Obstetrician | OR |
| **Combined Search** | (Midwifery care OR Maternal care OR Care) AND (Postpartum OR post-partum OR Perinatal OR Postnatal OR Parturition OR After childbirth OR After birth OR Following birth OR Puerperium) AND (General Practitioners OR Midwife OR Maternal and Child Health Nurses OR Obstetrician) | AND, OR, Parentheses |
| **Search Modifiers** | Truncation and Boolean operators were applied to maximise sensitivity (e.g., "postnatal*" to capture "postnatal", "postnatally") | * , AND, OR |

Table S1: Search Strategy Components
